# Integrating Network Pharmacology and RT-qPCR Analysis to Investigate the Mechanisms Underlying ZeXie Decoction-Mediated Treatment of Non-alcoholic Fatty Liver Disease

**DOI:** 10.3389/fphar.2021.722016

**Published:** 2021-09-09

**Authors:** Jiashuo Wu, Fangqing Zhang, Haonan Ruan, Xiaoyan Chang, Jingxun Wang, Zhuangzhuang Li, Weiyi Jin, Yue Shi

**Affiliations:** ^1^ Institute of Medicinal Plant Development, Chinese Academy of Medical Sciences and Peking Union Medical College, Beijing, China; ^2^ College of Public Health, Hebei Medical University, Shijiazhuang, China

**Keywords:** ZeXie decoction, network pharmacology, RT-qPCR analysis, non-alcoholic fatty liver disease, integrating strategy

## Abstract

ZeXie Decoction (ZXD) is a traditional Chinese medicine composed of *Alisma orientalis* (Sam.) Juzep. and *Atractylodes macrocephala* Koidz. ZXD has been widely used to treat non-alcoholic fatty liver disease (NAFLD). The mechanistic basis for the pharmacological activity of ZXD, however, remains poorly understood. In this study, we used a network pharmacology approach and investigated the association between ZXD and NAFLD. We identified the active ingredients of ZXD and screened the potential targets of these ingredients, after which a database of relevant NAFLD-related targets were constructed and several enrichment analyses were performed. Furthermore, the ethanol and aqueous extracts of ZXD were prepared and experimental pharmacology validation was conducted using RT-qPCR of the non-alcoholic fatty liver disease (NAFLD) model in Sprague-Dawley (SD) rats. As a result, a herb-compound-target-pathway network model was developed, and HMGCR, SREBP-2, MAPK1, and NF-*κ*Bp65 targets were validated. The gene expression results of these four targets were consistent with those of the network pharmacology prediction. Using an integration strategy, we revealed that ZXD could treat NAFLD by targeting HMGCR, SREBP-2, MAPK1, and NF-*κ*Bp65.

## Introduction

Rising obesity rate has partially been linked to the increasing prevalence of non-alcoholic fatty liver disease (NAFLD), characterized by hepatic steatosis unrelated to alcohol consumption (Than and Newsome, 2015). More than 25% of people suffer from NAFLD worldwide. A significant cause of human morbidity, NAFLD, can progress to severe conditions, including fibrosis, cirrhosis, and hepatoma (Tandon, 2013; Angulo et al., 2015). Treatment options for NAFLD are limited and are centered mainly around bariatric surgery and lifestyle changes.

Traditional Chinese medicine formulations (TCMFs) are traditional medicines that are used clinically for many years. ZeXie Decoction (ZXD) is one of the TCMFs composed of *Alismatis Rhizoma* (ZX, the rhizome of Alisma orientale (Sam.) Juzep.) and *Atractylodis Macrocephalae Rhizoma* (BZ, the rhizome of Atractylodes macrocephala Koidz.) in the ratio of 5:2 on a dry weight basis. ZX contains bioactive triterpenoid, including alisol A, alisol B, alisol A 23-acetate, alisol A 24-acetate, alisol B 23-acetate and so forth. BZ contains bioactive triterpenoid, including atractylenolide I, atractylenolide II, atractylenolide III and so forth (Hoang et al., 2016; Liu et al., 2019). Several mechanistic studies have found that ZXD, ZX, BZ, and other active derivatives could treat NAFLD through the modulation of insulin, and PI3K/AKT/NF-*κ*B and PPAR pathways, thereby suppressing inflammation and lowering lipid levels in treated individuals (Dan et al., 2011; Song et al., 2014; Li et al., 2016; Bi et al., 2017; Tang et al., 2017; Wu et al., 2018). ZXD is a promising complementary therapy for the treatment of NAFLD.

TCMFs are typically designed as multi-component multi-target pharmaceutical agents, in contrast to modern drugs typically designed to target a single protein (Zhang et al., 2019). Presently, the molecular mechanisms underlying the efficacy of many TCMFs remain uncertain. In the past, experimental model systems were leveraged for pharmacological studies of TCMFs. However, developments in systems biology have led to more widespread use of network pharmacology-based analyses of traditional medicine preparations (Lee et al., 2018; Zuo et al., 2018; Huang et al., 2020). Network pharmacology tools offer an ideal opportunity to understand the complex mechanisms through which TCMFs function *in vivo.*

**FIGURE 1:**
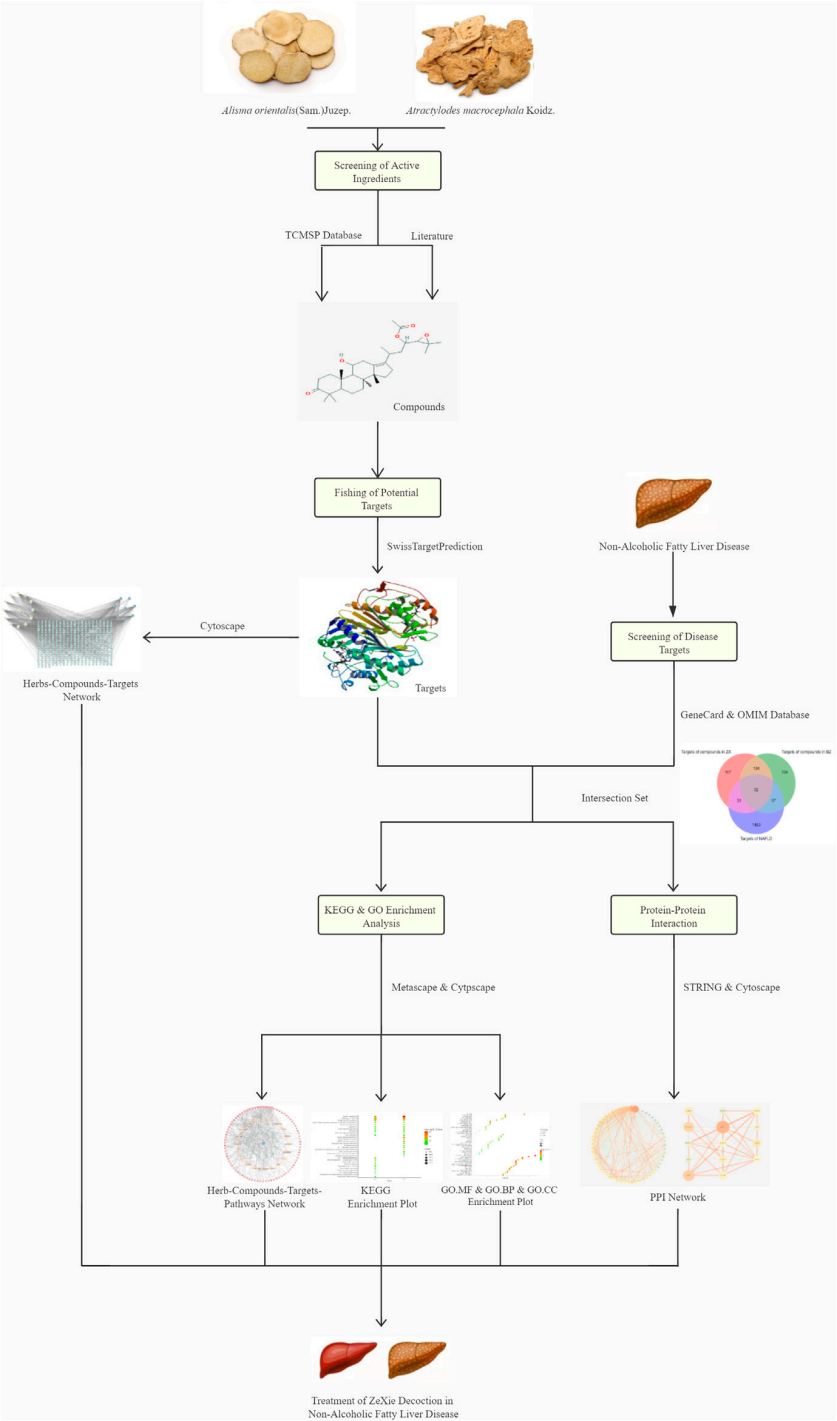
The framework of network pharmacology.

In our previous work (Chang et al., 2021), we studied ethanol and aqueous extracts of ZXD using ultra-performance liquid chromatography coupled with mass spectrometry to identify the major components of ZXD. Based on this work, published literature, and database sources, we developed a herb-compound-target-pathway network model and performed enrichment analysis to investigate the relationship between ZXD and NAFLD treatment. The network pharmacology framework is depicted in Figure 1. Basing on the non-alcoholic fatty liver disease (NAFLD) model in Sprague-Dawley (SD) rats, the prediction of the mechanism was validated by RT-qPCR analysis *in vivo*.

## Materials and Methods

### Experimental Animals

A total of 72 male SD rats weighting 200 ± 20 g (Beijing Vital River Laboratory Animal Technology Co. Ltd.) were housed in a room at a constant temperature of 23 ± 3°C, a constant humidity of 50 ± 10%, under a 12 h light/dark cycle. The rats were fed UV-disinfected fodder and drinking water. The animal experiment was approved by the Laboratory Animal Center, Institute of Medicinal Plant Development, Chinese Academy of Medical Sciences and Peking Union Medical College. The rats were adaptively bred for 1 week in the facility before the experiments.

### Reagents

The dried rhizomes of *ZX* (Batch number: DD6081) and *BZ* (Batch number: DD8061) were purchased from Beijing Huamiao Pharmaceutical Co., Ltd. (Beijing, China). Their qualities were in accordance with the Chinese Pharmacopoeia. The specimens of ZX and BZ have been preserved in the Institute of Medicinal Plant Development, Chinese Academy of Medical Sciences and Peking Union Medical College.

PrimeScript RT reagent kit, TRIzol reagent, and DNA marker were purchased from TaKaRa Bio (Beijing, China). The total cholesterol (TC), triglycerides (TG), aspartate aminotransferase (AST), alanine aminotransferase (ALT) assay kit were purchased from Beijing Solarbio Science and Technology Co. Ltd (Beijing, China). Urethane, pioglitazone hydrochloride (PH) and paraformaldehyde were purchased from Macklin Reagent Company (Shanghai, China).

### Sample Preparation

The samples of ZXD were prepared by combining ZX and BZ in a 5:2 ratio (w/w). The samples were crushed and refluxed thrice with 10-times the volume of 95% ethanol for 2 h. The filtrates were then pooled and dried using a rotary evaporator under vacuum, yielding 14.29% ethanol extract.

### Qualitative Analysis

The comparative study of the ethanol and aqueous extracts of ZXD using UPLC-DAD-Q-TOF-MS was performed in our previous study to simultaneously identify the major chemical components in these two extracts (Chang et al., 2021). This analysis was conducted using an Waters ACQUITY UPLC system with a binary solvent delivery system and an autosampler. An Waters Acquity UPLC HSS T3 column (1.7 μm, 2.1 mm × 100 mm; Waters, MA, United States) was used to conduct this analysis at 30°C. Acetonitrile (A) and formic acid in water (0.1%, v/v) (B) were used as the mobile phase in this analysis, with 0.2 ml/min being selected as the flow rate. A linear gradient elution approach was used with the following settings: 0–10 min, 0–1% A; 10–15 min, 1–3% A; 15–20 min, 3–10% A; 20–25 min, 10–15% A; 25–30 min, 15–20% A; 30–35 min, 20–30% A; 35–40 min, 30–60% A; 40–45 min, 60–80% A; 45–50 min, 80–90% A; 50–55 min, 90–100% A. A 2 µL injection volume was used for all analysis, with 15°C as the injection temperature.

A Q-TOF analyzer in the SYNAPT G2 HDMS system (Waters, Manchester, United Kingdom) was used for tandem MS analyses. Positive ionization mode was used to acquire ESI spectra in the m/z 50–1,200 mass range with the following settings: a 120°C source temperature; 3.0 kV as the chosen capillary voltage; a desolvation gas temperature of 450°C and a gas flow rate of 800 L/h; a 40.0 V sampling cone voltage and a 50 L/h cone gas rate; 10–40 eV as the collision energy. The Masslynx 4.1 software package was used to acquire all data.

### The Study of Network Pharmacology

#### Bioactive Ingredient Identification

The widely used Traditional Chinese Medicine System Pharmacology database and Analysis Platform (Ru et al., 2014) (TCMSP, https://tcmspw.com/tcmsp.php) was utilized to screen the active ingredients within ZX, BZ, and ZXD. In total, 101 herbal ingredients were identified using the keywords “*Alisma Orientale* (Sam.) Juzep.” and “*Atractylodes Macrocephala* Koidz.”. The potential active ingredients were then selected using an oral bioavailability (OB) threshold of ≥30% and a drug-likeness (DL) threshold of ≥0.18 (Leeson and SPRINGTHORPE, 2007; Xu et al., 2012). In addition, any compounds in ZXD, ZX, or BZ that have previously been reported to be highly pharmacologically active were also included in this analysis (Wu et al., 2021). Two-dimensional (2D) structures of all compounds were obtained from the PubChem database (https://pubchem.ncbi.nlm.nih.gov/) and used for target prediction efforts.

#### Target Prediction

TheSwissTargetPrediction platform (David et al., 2013) (http://www.swisstargetprediction.ch/index.php) was utilized to identify the putative targets of the compounds retrieved from the PubChem database. The Swiss library contains over 3,000 proteins and 370,000 active ingredients, making it ideal for such efforts. Files of the identified bioactive ingredients with the suffix “SDF” were used as inputs for the SwissTargetPrediction platform, and probable macromolecular targets of these compounds were identified based on structural similarity. Only the SwissTargetPrediction platform was used for this predictive analysis to ensure the clarity of results. The species was set to *Homo sapiens* for this analysis and all subsequent analyses.

#### NAFLD-Related Target Identification

Annotated and predicted NAFLD-related targets were identified using the Online Mendelian Inheritance in Man (OMIM, https://omim.org/) (Hamosh et al., 2002) and GeneCards (https://www.genecards.org/) (Stelzer et al., 2016) databases. We used “Non-alcoholic fatty liver” and “Non-alcoholic fatty liver disease” as search terms and integrated the search results to develop a NAFLD-specific disease target database. NAFLD-related targets that were also identified as putative targets of ZXD bioactive ingredients were retained for further analysis.

#### Network Construction and Enrichment Analyses

Cytoscape 3.7.2 (Shannon et al., 2003) (https://cytoscape.org/) was used to construct a herb-compound-target network, with nodes corresponding to herbs, compounds, and targets and edges representing between-node interactions. Node sizes were scaled based on degree values determined using Cytoscape Network Analyzer such that smaller nodes were associated with smaller degree values.

The potential functional roles of targets overlapping between ZXD and NAFLD target lists were evaluated *via* Metascape (Zhou et al., 2019) (https://metascape.org/), Kyoto Encyclopedia of Genes and Genomes (KEGG) pathway analyses, and Gene Ontology (GO) enrichment analyses. All genes were used as the enrichment background for KEGG analyses, with significantly enriched terms meeting the following criteria: count ≥3, *p* < 0.01, enrichment factor >1.5. These terms were grouped and clustered based on the membership similarity. For GO analyses, the molecular functions (MFs), biological processes (BPs), and cellular components (CCs) for which these targets were enriched were identified and visualized using enrichment plots.

The ZX and BZ herb-compound-target-pathway networks were then plotted based on the results of the above enrichment analyses.

#### Protein-Protein Interaction Analysis

Protein-protein interaction (PPI) analysis was conducted to identify which ZXD targets are most crucial in the context of disease pathology. STRING 11.0 (Szklarczyk et al., 2017) (https://string-db.org/) database was used to conduct PPI analysis of overlapping ZXD and NAFLD targets and KEGG pathway analysis. For the PPI analysis, active interaction sources were text mining, experiments, databases, co-expression, neighborhood, gene fusion, and co-occurrence. The analysis range was only set to input proteins, and output obtained in the form of files with the suffix “TSV” was visualized using Cytoscape 3.7.2; NetworkAnalyzer was used to assess the degree and combined scores.

All macromolecular targets were retrieved from the Uniprot database (Alex, 2019) (https://www.uniprot.org/) to confirm their gene symbols. The plots described above were designed based on these symbols to ensure statistical normalization.

### Experimental Pharmacology Validation

#### Experimental Design

Seventy-four male SD rats were randomly divided into two groups according to their diets, including blank (n = 13) and model group (n = 61). The blank group rats were fed with normal chew diets (NCD, Beijing Hfk Bioscience Co., Ltd.), and the model group rats were fed with high-fat diets containing 41% fat (HFD, Beijing Hfk Bioscience Co., Ltd.) for 12 weeks to induce NAFLD. After 12 weeks of modeling, one rat liver tissue from each group was used for histopathological evaluation to validate the NAFLD model. Afterwards, a 4-weeks intervention period with ZXD and positive control drug started. Rats from model group were randomly and equally assigned to five subgroups: vehicle, positive control, low-dose, middle-dose and high-dose groups (n = 12 each). The vehicle group and positive control group rats were orally administered distilled water and 10 mg/kg/d PH, respectively. The low-dose, middle-dose and high-dose group rats were orally administered 100, 200 and 400 mg/kg/d ZXD, respectively. The doses of ZXD and PH were selected based on the previous studies (Hong et al., 2010; Gong et al., 2018; Yang et al., 2019). During the 4-week intervention period, the blank group rats were fed with NCD and the remaining five group rats were fed with HFD. Animals were given free access to feed and drink. The animal weights and food consumption were monitored daily. At the end of intervention period, animals were fasted for 16 h and then anesthetized with an intraperitoneal injection of urethane (20%, 1 g/kg). The biological samples (blood, liver and urine) were collected and stored at -80°C to conduct the histopathological evaluation and serum biochemical assay.

#### Histopathological Evaluation

##### Hematoxylin and Eosin (HE) Staining

Fresh liver tissues were fixed with 4% paraformaldehyde for 24 h. The fixed tissues were then embedded in paraffin, cut into thin slices and stained with HE. Light microscopy was used to determine the histopathological changes.

##### Oil Red O (ORO) Staining

Frozen liver tissues were cut into thin sections and then mounted on glass slides. The slides were fixed in pre-cold 4% paraformaldehyde and rinsed with distilled water. The rinsed slides were placed in isopropanol solution and then incubated in ORO solution for 10 min. Afterwards, the ORO solution was removed and the slides were rinsed with running tap water for 5 min. Light microscopy was used to determine the histopathological changes.

#### Serum Biochemical Assays

The blood samples were collected and left standing for 30 min. The samples were centrifuged for 10 min at 3,000 g to separate the serum for biochemical assays. The total cholesterol (TC), triglycerides (TG), aspartate aminotransferase (AST), alanine aminotransferase (ALT) levels were measured according to the kit instructions.

#### RT-qPCR

RT-qPCR was then performed to measure the hepatic expression of the predicted NAFLD-related genes. Total RNA was collected with the RNA isolation kit according to the instructions. The purity of the collected RNA was detected by the NanoDrop ND-2000 spectrophotometer, and the 260/280 ratios were calculated. PrimeScript™ RT reagent kit with gDNA eraser was used to carry out the reverse transcription reactions. The Realtime-PCR was performed on the ABI7500 fast system. The calculation of relative gene expression was based on the 2^−ΔΔCT^ method. The primers used in the experiments are listed in Table 1.

**TABLE 1:** PCR primer design.

Gene name	Forward primer (5′-3′)	Reverse primer (5′-3′)
HMGCR	TTG​CAC​GTC​TAC​AGA​AAC​TTC​ATA​C	CCT​GAC​CTG​GAC​TGG​AAA​CG
SREBP-2	TGT​GAC​CTG​CTA​CTG​TCG​CTA	AAC​ACC​TTG​CGG​TAT​GCT​G
MAPK	ATT​ACG​ACC​CGA​GTG​ACG​AG	CAA​AGT​GGA​TAA​GCC​AAG​AC
NF-*κ*B	CGA​CGC​ATT​GCT​GTG​CCT​TC	ATG​GTG​CTC​AGG​GAT​GAC​GTA​AAG

#### Statistical Analysis

Statistical analysis was performed using SPSS 23.0 software, and the results are presented as mean ± SD. One-way analysis of variance was used for comparison, and *p*-value＜0.05 was considered statistically significant. The data graphs were generated using GraphPad Prism 8.

## Results

### Active Ingredients of ZXD

In our initial search of the TCMSP database, we identified 101 active ingredients of potential relevance. Of these, we selected six ingredients in ZX (Z1 to Z6) and four ingredients in BZ (B4 to B7) based upon their OB and DL values. In addition, we selected nine ingredients (Z7 to Z12 and B1 to B3) that have previously been reported to exhibit a high degree of pharmacological activity in ZXD preparations (Lin, 2012; Xu et al., 2016a; Chao et al., 2016; Song et al., 2017; Xu et al., 2018; Ho et al., 2019; Wu et al., 2021). These ingredients were numbered Z1-Z12 and B1-B7, and compound CID was confirmed with the Pubchem database (Table 2).

**TABLE 2:** Information of ingredients in ZXD.

Id	CID	Chemical name	Source	Literature
Z1	14036813	Alisol C Monoacetate	ZX	Li et al. (2016); Chang et al. (2021)
Z2	101306923	Alisol C	ZX	Li et al. (2016); Chang et al. (2021)
Z3	14036811	Alisol B 23-Acetate	ZX	Szklarczyk et al. (2017); Chang et al. (2021)
Z4	15558620	Alisol B	ZX	Alex (2019); Chang et al. (2021)
Z5	222284	Sitosterol	ZX	Chang et al. (2021)
Z6	5283469	1-Monolinolein	ZX	Chang et al. (2021)
Z7	15558616	Alisol A	ZX	Gong et al. (2018)
Z8	74344393	Alisol A 24-Acetate	ZX	Szklarczyk et al. (2017); Wu et al. (2018)
Z9	70690607	Alisol A 23-Acetate	ZX	Hong et al. (2010)
Z10	102004739	25-Anhydroalisol F	ZX	Bi et al. (2017)
Z11	76310822	Alisol F	ZX	Bi et al. (2017)
Z12	70688546	Alisol M 23-Acetate	ZX	Yang et al. (2019)
B1	5321018	Atractylenolide I	BZ	Hong et al. (2010); Tang et al. (2017)
B2	14448070	Atractylenolide II	BZ	Hong et al. (2010)
B3	155948	Atractylenolide III	BZ	Xu et al. (2016a)
B4	132941086	14-Acetyl-12-Senecioyl-2E,8Z,10E-Atractylentriol	BZ	Chang et al. (2021)
B5	73170	*α*-Amyrin	BZ	Chang et al. (2021)
B6	15976101	(24S)-24-Propylcholesta-5-Ene-3*β*-Ol	BZ	Chang et al. (2021)
B7	14448075	8β-Ethoxy Atractylenolide Ⅲ	BZ	Chang et al. (2021)

The CID of compound was obtained from PubChem database.

### Identification of ZXD and NAFLD Targets

**FIGURE 2:**
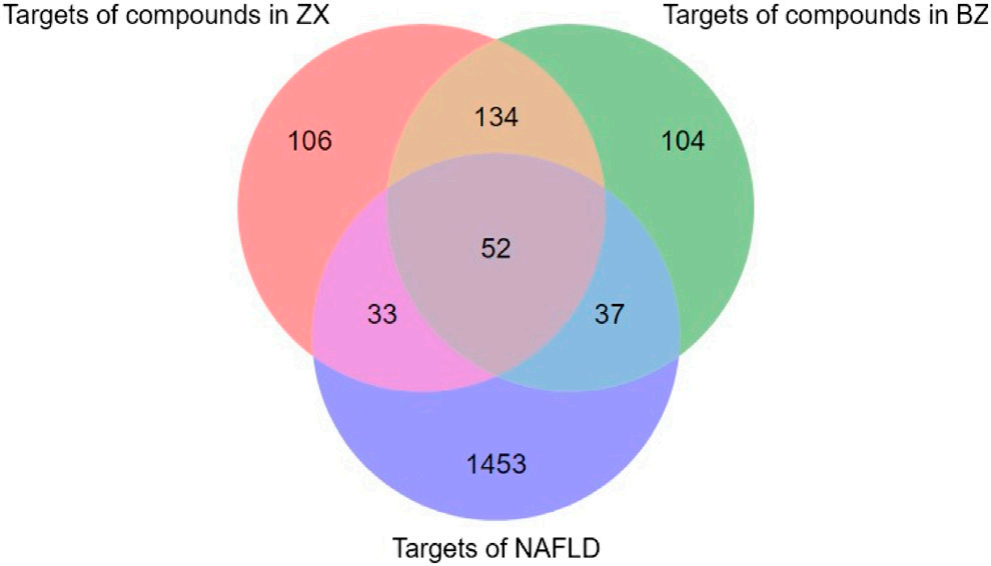
The venn diagram of targets in ZX, BZ and NAFLD. Totally 1575 NAFLD-related targets were identified, of which 122 targets overlapped with our list of ZXD targets.

These 19 active compounds were next introduced into the Swiss platform to identify putative macromolecular targets. We identified 325 ZX targets and 327 BZ targets, and 186 overlapping targets (Supplementary Table 1). In addition, the OMIM and GeneCards databases were used to identify 1575 NAFLD-related targets, of which 122 targets overlapped with our list of ZXD targets (Supplementary Table 2) (Figure 2). These overlapping targets were retained, and corresponding Uniprot IDs were determined and used to identify corresponding gene symbols for each target.

**FIGURE 3:**
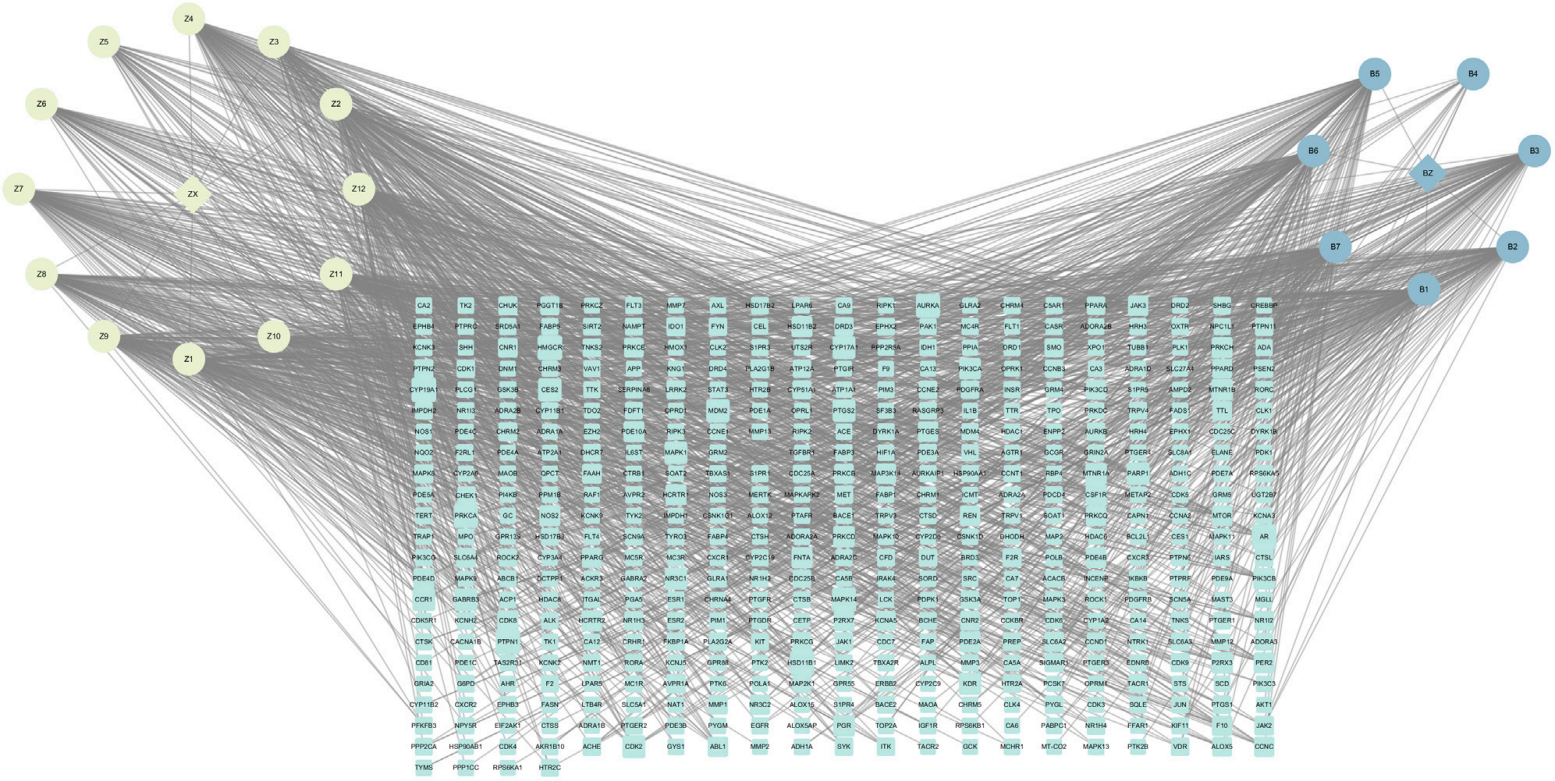
The herbs-compounds-targets network of 143 nodes. These 19 active ingredients and 122 targets were incorporated into a herbs-compounds-targets network wherein node size was scaled according to the degree values. The light-yellow nodes represent ZX and its compounds, the light blue nodes represent BZ and its compounds and the light green nodes represent the gene symbols. The size of node is proportional to the degree value and the edges represent interactions between the nodes.

These 19 active ingredients and 122 targets were incorporated into a herbs-compounds-targets network wherein node size was scaled according to the degree values (Figure 3). The targets with the highest degree values included HMGCR, MAPK1, and SREBP-2, suggesting that these targets may play critical roles in the context of NAFLD treatment.

### Functional Enrichment and PPI Network Analyses

**FIGURE 4:**
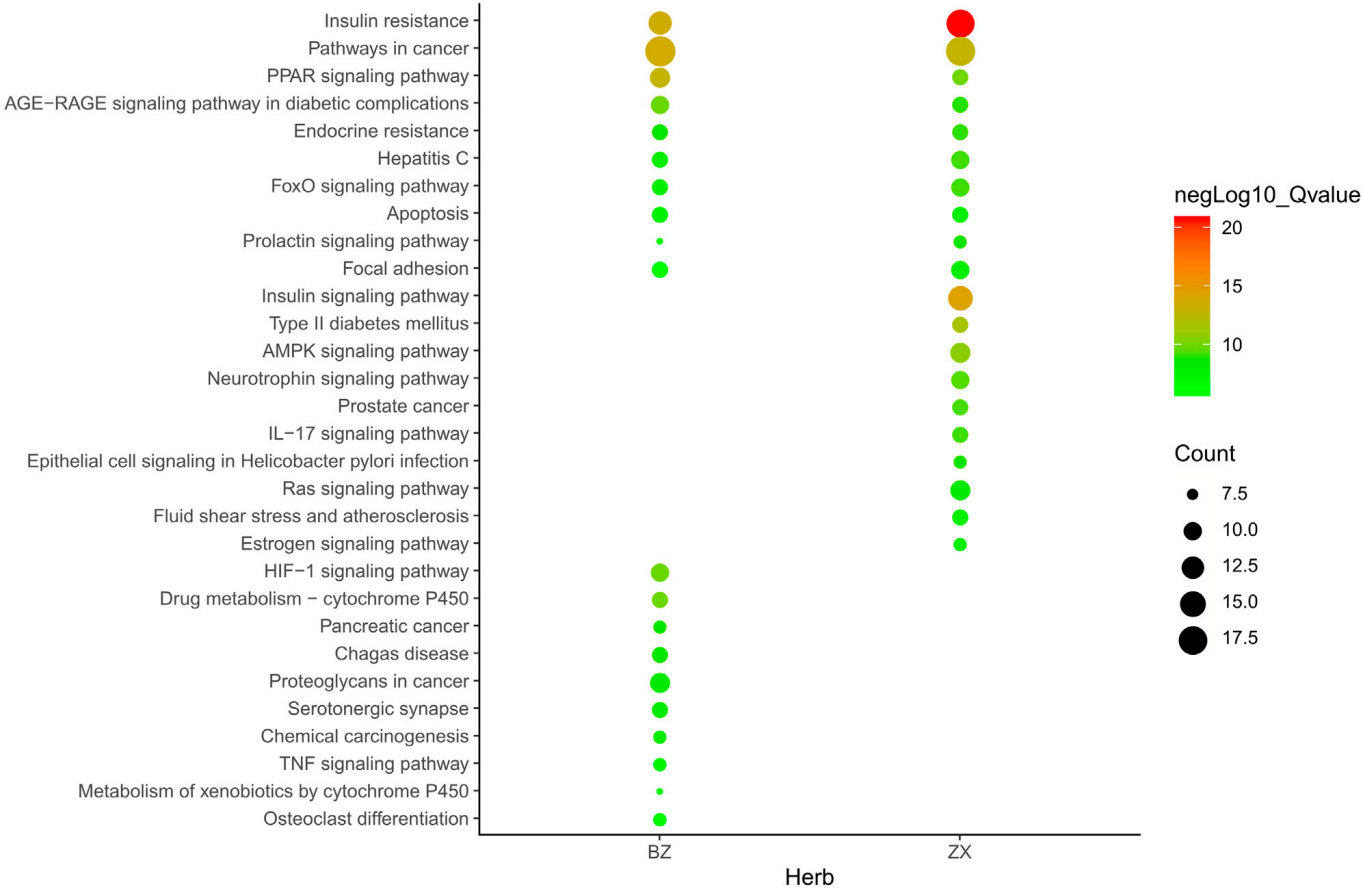
The Enrichment plot of KEGG pathway analysis. The size and color of nodes represent the count and–log10(q) values of pathways, respectively.

**FIGURE 5:**
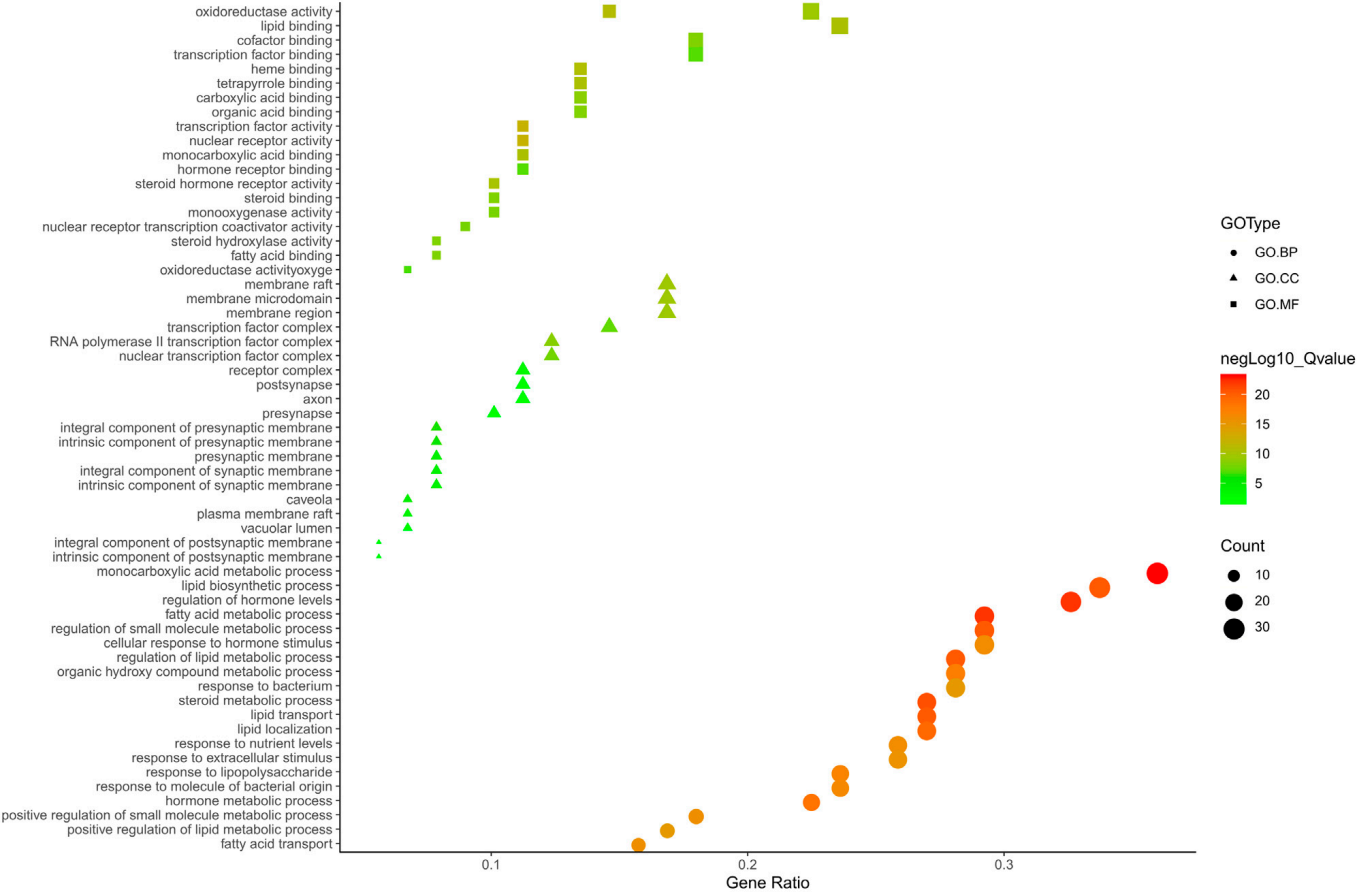
The GO. MF, GO. BP and GO. CC enrichment analysis plot of ZX. The size and color of nodes represent the count and–log10(q) values of molecular functions, biological processes and cellular components items, respectively.

**FIGURE 6:**
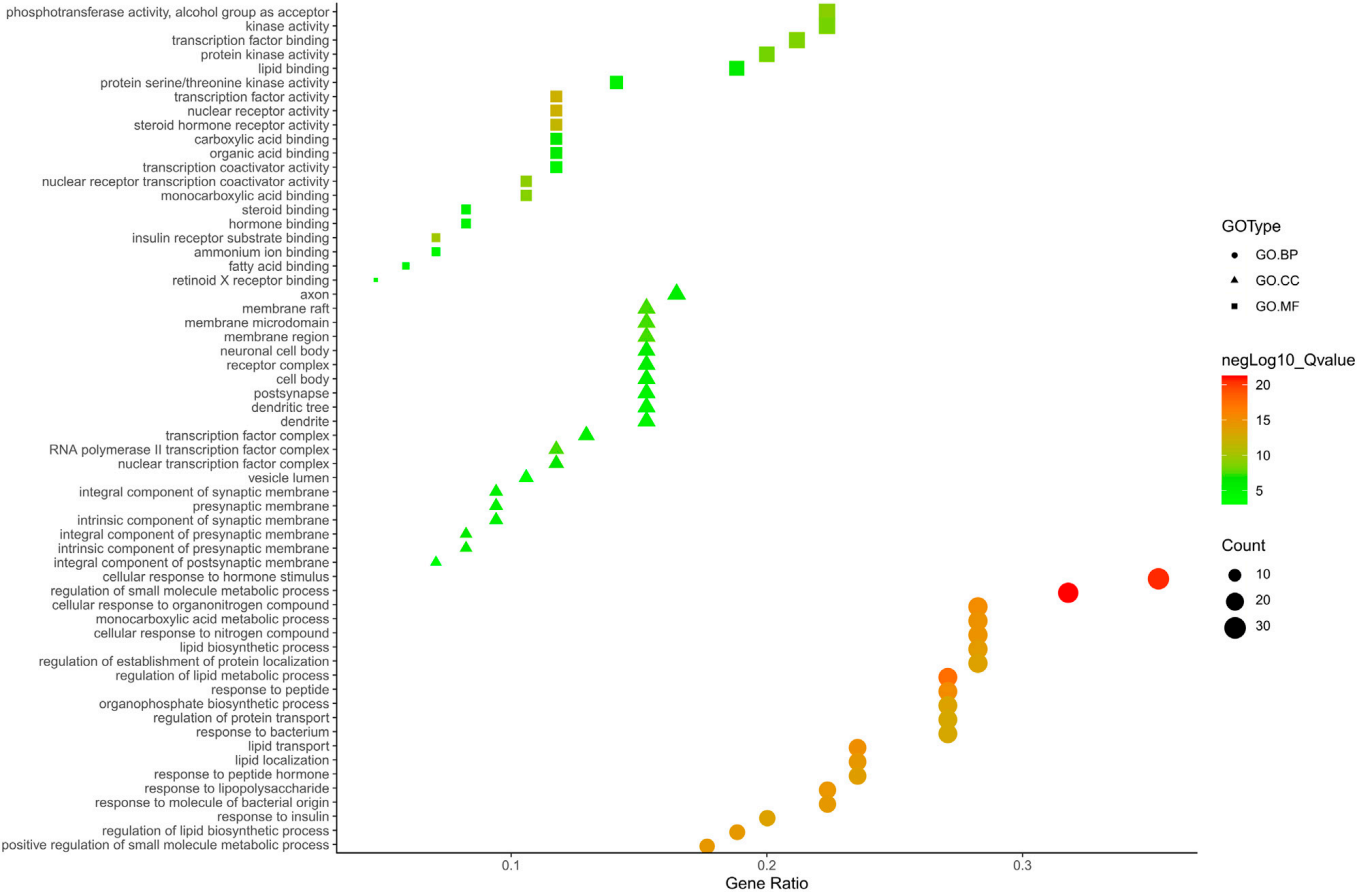
The GO. MF, GO. BP and GO. CC enrichment analysis plot of BZ. The size and color of nodes represent the count and–log10(q) values of molecular functions, biological processes and cellular components items, respectively.

Metascape platform was used to carry out KEGG pathway and GO enrichment analyses of the 122 overlapping ZXD and NAFLD targets. Results were ranked based on ascending q-values, corresponding to the degree of correlation with these targets, and the top 20 of these enriched pathways were plotted (Supplementary Table 3) (Figures 4–6). These targets were enriched for the lipid metabolism GO BP, suggesting that this pathway may be linked to the pharmacological activity of ZXD. Other identified targets associated with this pathway were also retained for further analysis (Supplementary Table 4).

**FIGURE 7:**
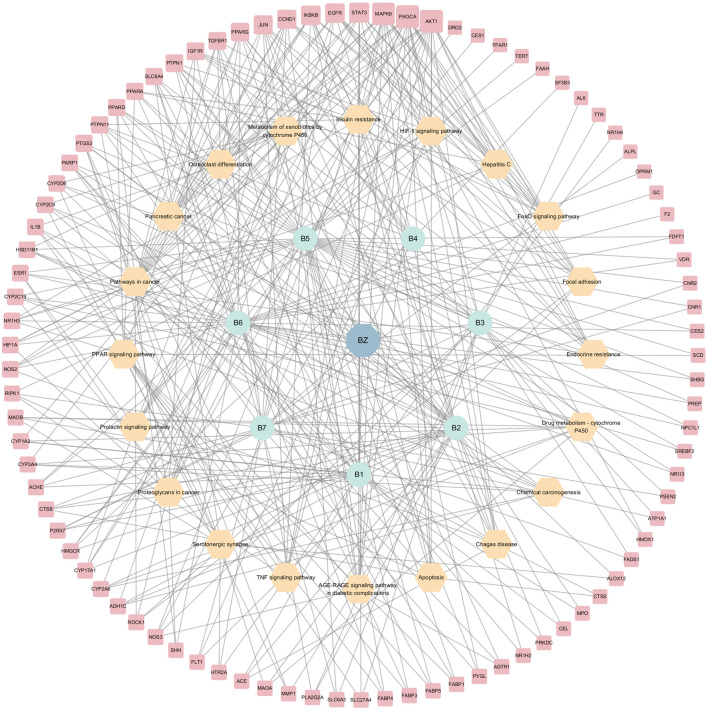
The herb-compounds-targets-pathways network of BZ. The light red nodes represent gene symbols, the light-yellow nodes represent pathways, the light green nodes represent compounds and the light blue nodes represent BZ. The size of node of gene symbol is proportional to the degree value and the edges represent interactions between the nodes.

**FIGURE 8:**
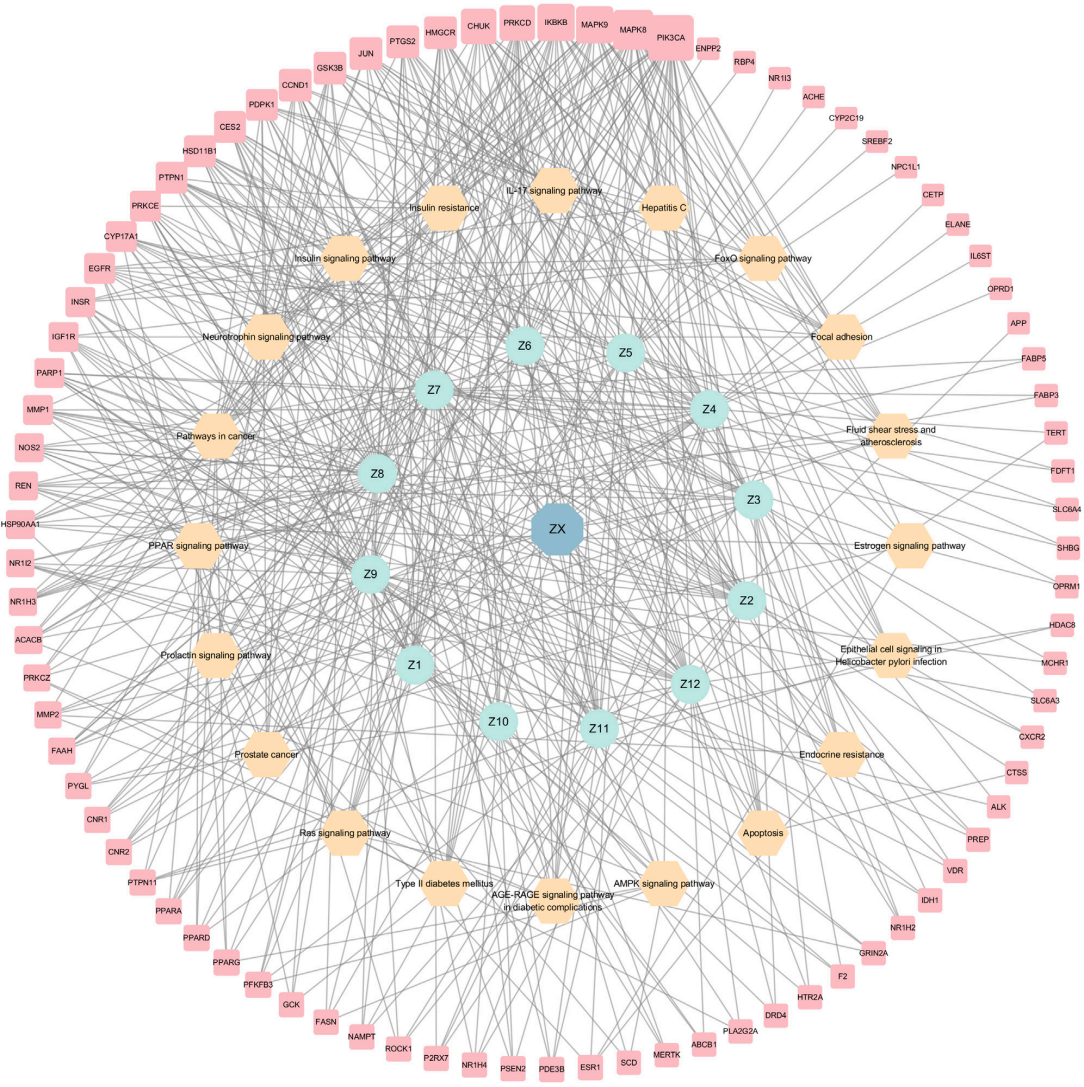
The herb-compounds-targets-pathways network of ZX. The light red nodes represent gene symbols, the light-yellow nodes represent pathways, the light green nodes represent compounds and the light blue nodes represent ZX. The size of node of gene symbol is proportional to the degree value and the edges represent interactions between the nodes.

KEGG analyses revealed that insulin-related signaling and PI3K/AKT/NF-*κ*B pathways are closely associated with ZXD-mediated NAFLD treatment. Using these results, we constructed a herb-compound-target-pathway network (Figures 7, 8).

**FIGURE 9:**
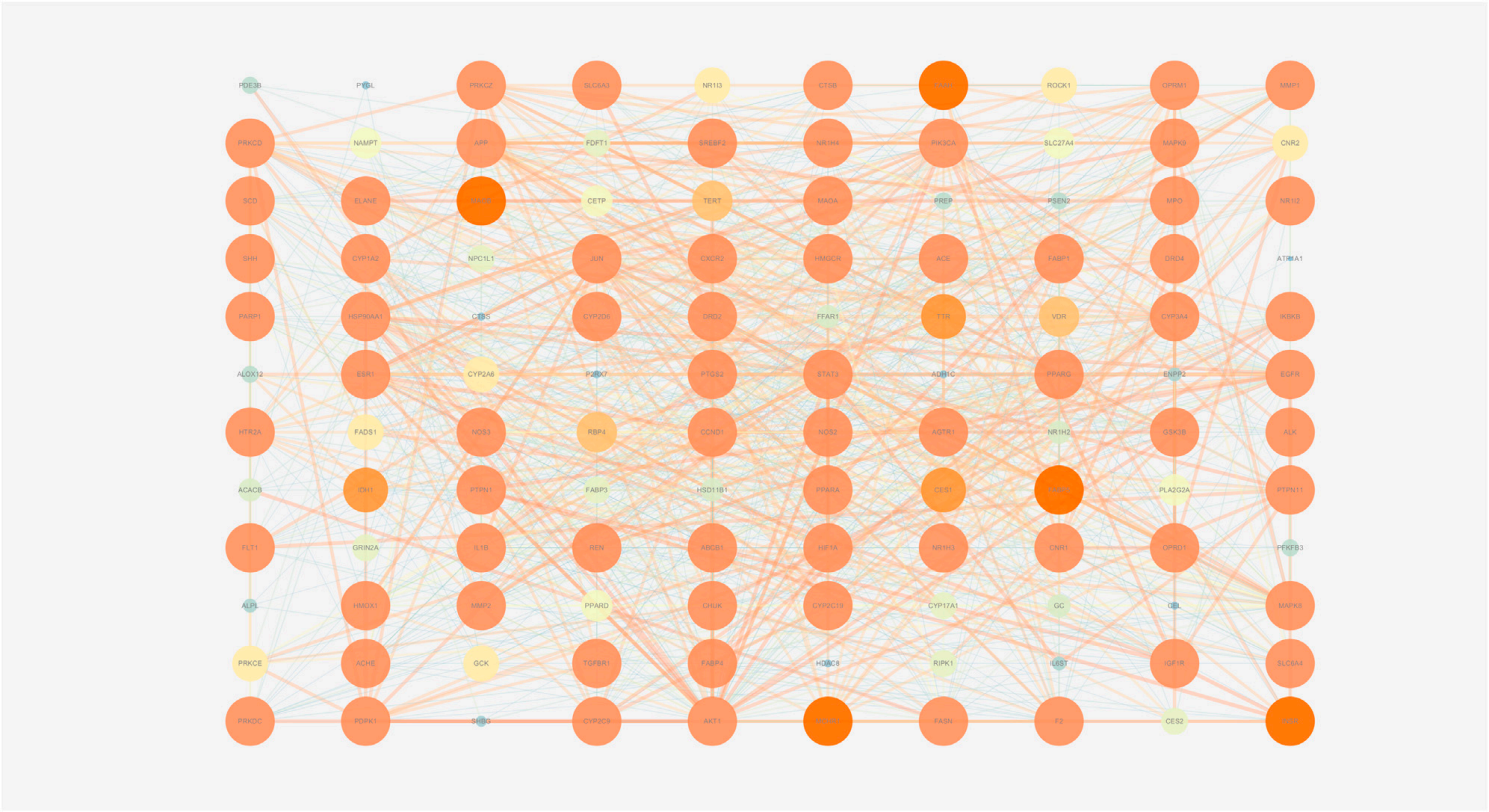
The PPI network of all matched targets. The size of nodes represents their degree values and the width of edges represents interactions between the nodes. The color represents their degree values and combined scores, which changes from blue to orange represent values from low to high.

**FIGURE 10:**
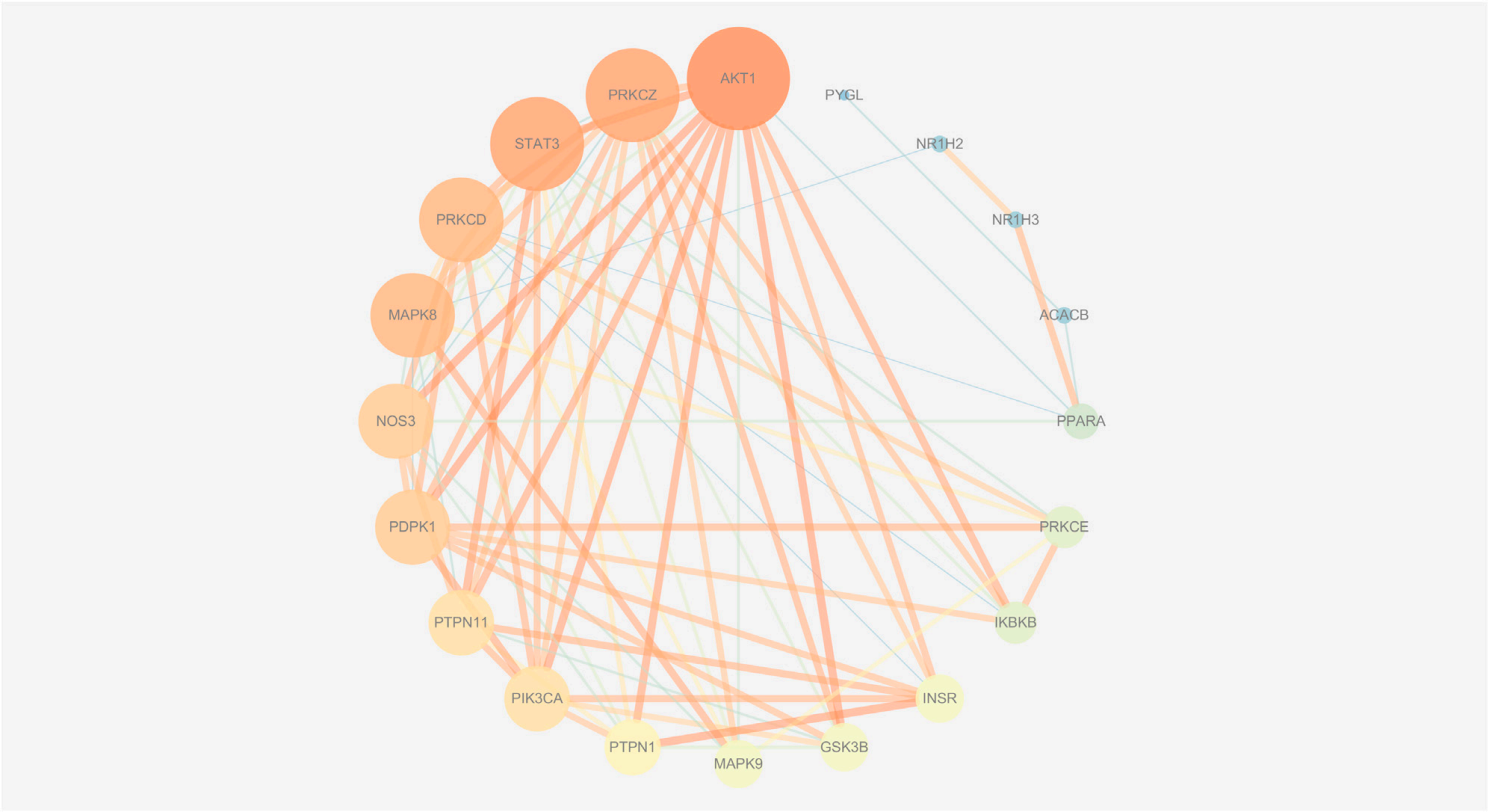
The PPI network of insulin-related targets. The size of nodes represents their degree values and the width of edges represents interactions between the nodes. The color represents their degree values and combined scores, which changes from blue to orange represent values from low to high.

To explore the interactions among the above targets in-depth, we constructed a PPI network using the STRING database with a medium confidence interaction threshold (0.4). Targets were then scored based on these predicted interactions and ranked in ascending order, retaining the targets that exhibited a combined score of ≥0.90 for further analysis (Supplementary Table 5). The degree values, centrality, and other parameters associated with these targets were then calculated using the Cytoscape Network Analyzer tool (Supplementary Table 6; Figures 9, 10). The data revealed precise, relevant interactions between these targets and highlighted the PI3K/AKT/NF-*κ*B pathway as an essential target worthy of further investigation in the context of NAFLD treatment using ZXD. When all 122 overlapping targets were plotted, the resultant data also suggested that mitogen-activated protein kinases (MAPKs), fatty acid-binding proteins (FABPs), and matrix metalloproteinases (MMPs) may warrant future investigation in a therapeutic context.

### Experimental Validation of Network Pharmacology

**FIGURE 11:**
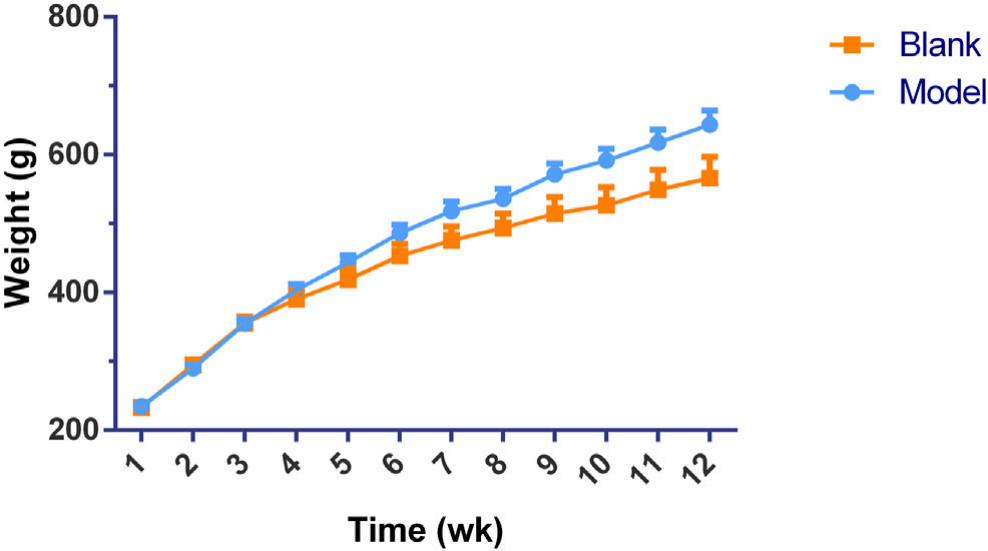
Change in body weight during the 12-week modeling period.

**FIGURE 12:**
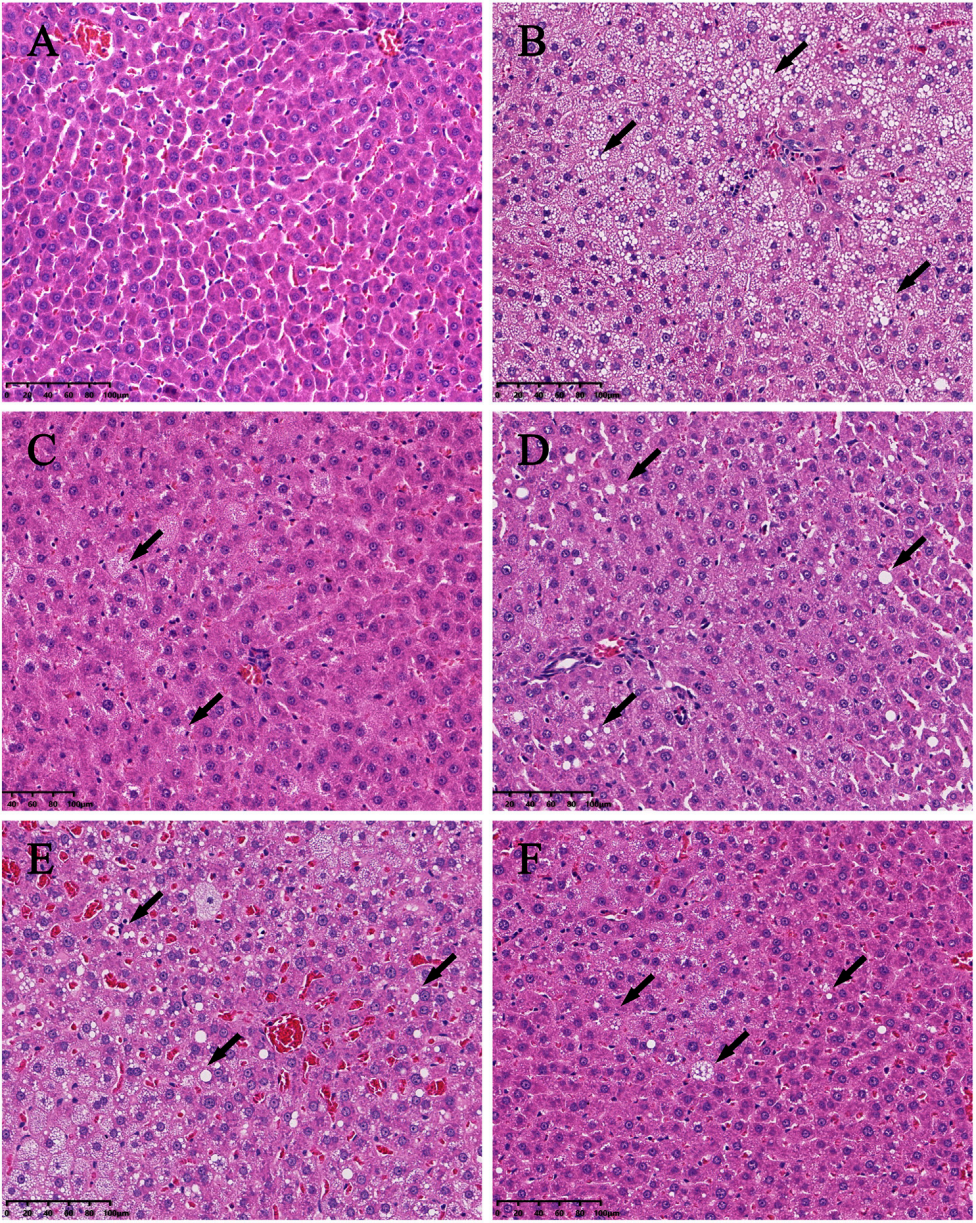
Histopathologic evaluation of liver tissues by HE staining (200×). **(A)** blank; **(B)** model; **(C)** positive control; **(D)** high-dose; **(E)** middle-dose; **(F)** low-dose. The lipid drops are indicated by arrows.

**FIGURE 13:**
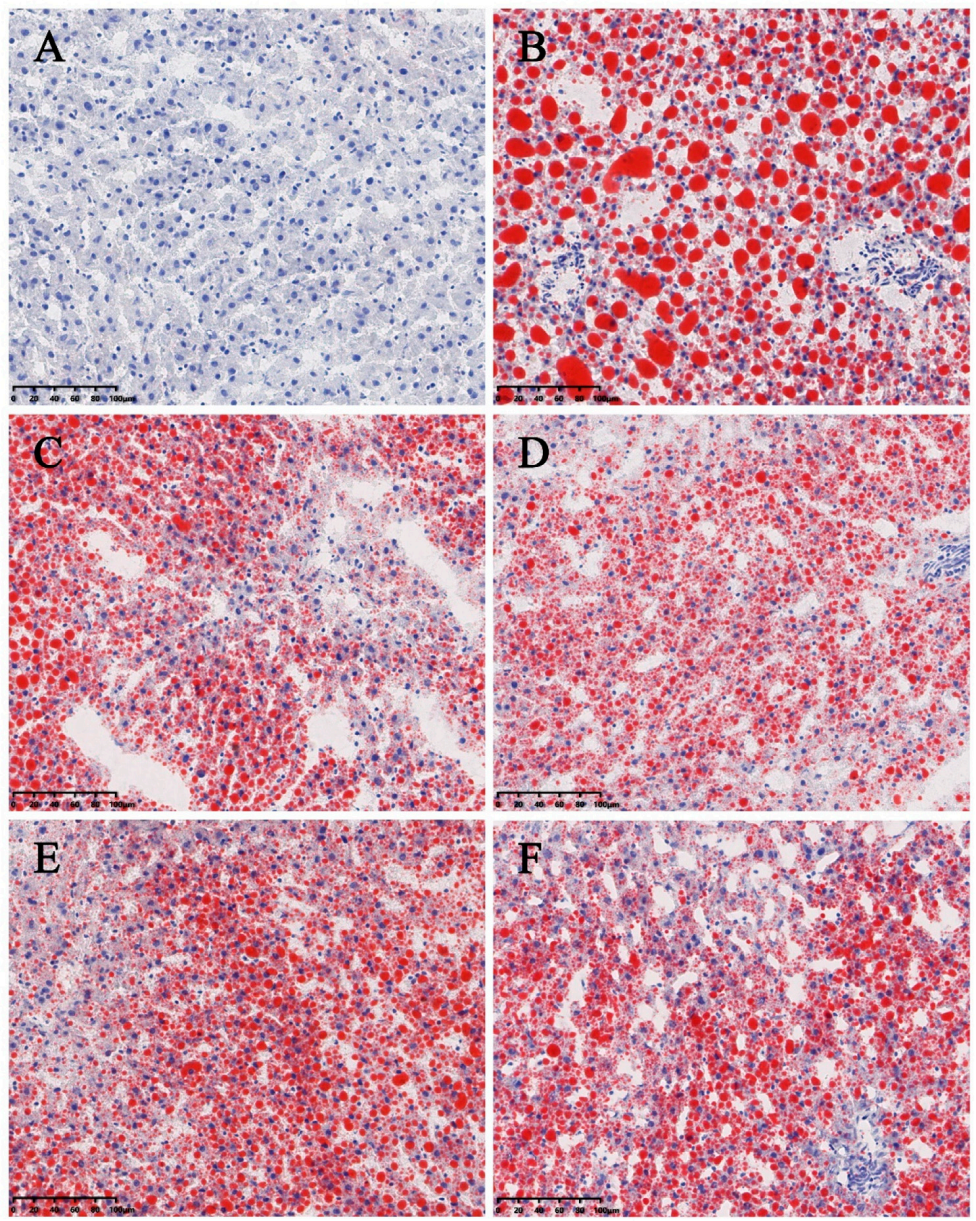
Histopathologic evaluation of liver tissues by ORO staining (200×). **(A)** blank; **(B)** model; **(C)** positive control; **(D)** high-dose; **(E)** middle-dose; **(F)** low-dose. Compared with the model group rats, the treated group rats reduced accumulation of lipid droplet remarkably.

**FIGURE 14:**
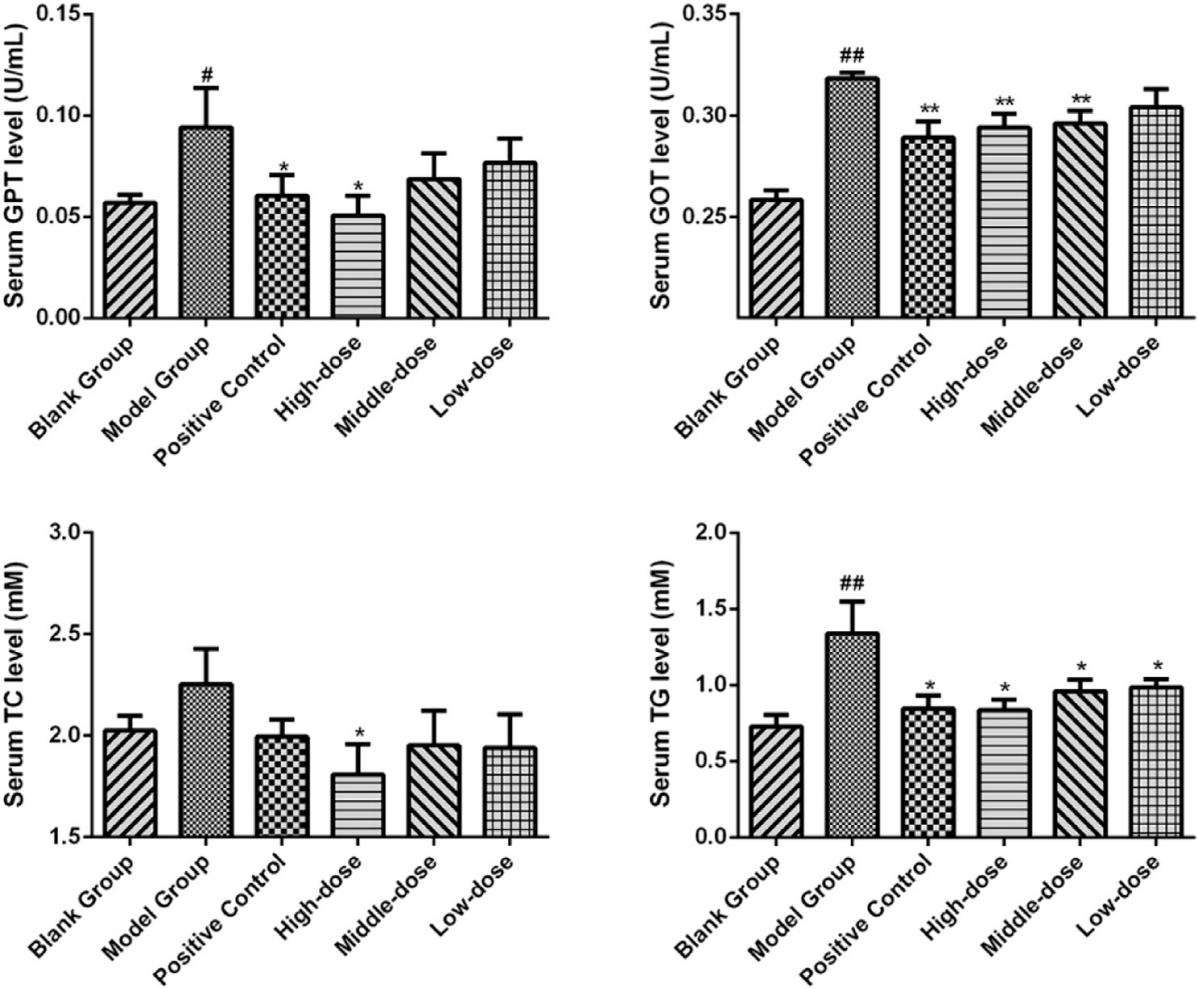
The serum biochemical parameter of rats after the 4-weeks intervention period. *p < 0.05 and **p < 0.01 vs. model group rats; #*p* < 0.05 and ##*p* < 0.01 vs. blank group rats.

Significant differences (*p* < 0.01) in body weights were observed between the model and blank group rats that were fed HFD and NCD for 12 weeks, respectively (Figure 11). Furthermore, the HE-stained liver tissues of the model group rats revealed significant differences compared with the tissues of the blank group rats (Figure 12 A-B; Figures 13A,B). Specifically, liver tissue sections from the blank group rats had intact, clear boundaries; The hepatocytes had a cord-like arrangement and were radially distributed around the central vein, and the cell nuclei was clearly visible. In contrast, swelling of hepatocyte, deposition of big lipid drops and hepatic ballooning degeneration were observed in liver tissue sections from the model group rats. The differences implied that the NAFLD modeling was successful. Afterwards, we treated NAFLD rats with the ethanol extract of ZXD across a range of concentrations (100, 200 and 400 mg/kg/d, respectively) for 4 weeks, and PH (10 mg/kg/d) was used as a positive control. We observed significant differences in the histopathological evaluation (Figure 12 C-F; Figures 13C–F) and serum biochemical assay (Figure 14) data of different groups. The curative effect of ZXD on NAFLD was found to be dose-dependent in rats at concentrations 100, 200 and 400 mg/kg/d.

**FIGURE 15:**
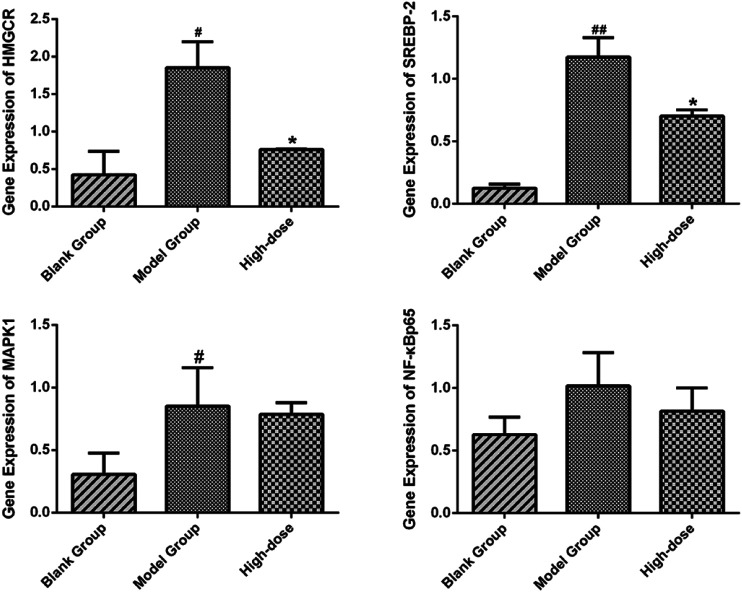
Expression of SREBP-2, HMGCR, NF-*κ*B and MAPK1 in SD rats. *p < 0.05 and **p < 0.01 vs. model group rats; #*p* < 0.05 and ##*p* < 0.01 vs. blank group rats.

**FIGURE 16:**
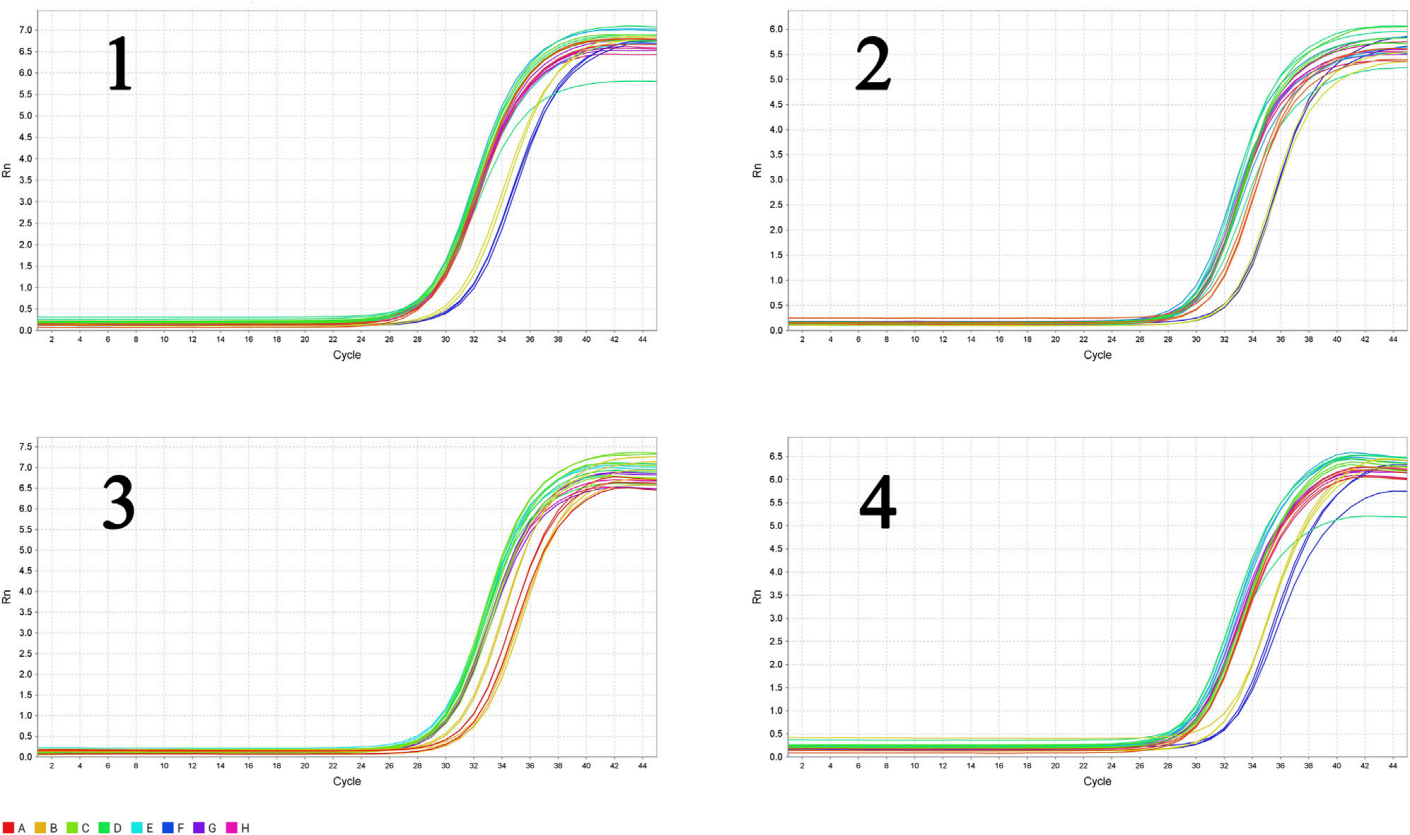
The amplification curve of the four major genes (1, SREBP-2; 2, HMGCR; 3, MAPK1; 4, NF-*κ*B).

**FIGURE 17:**
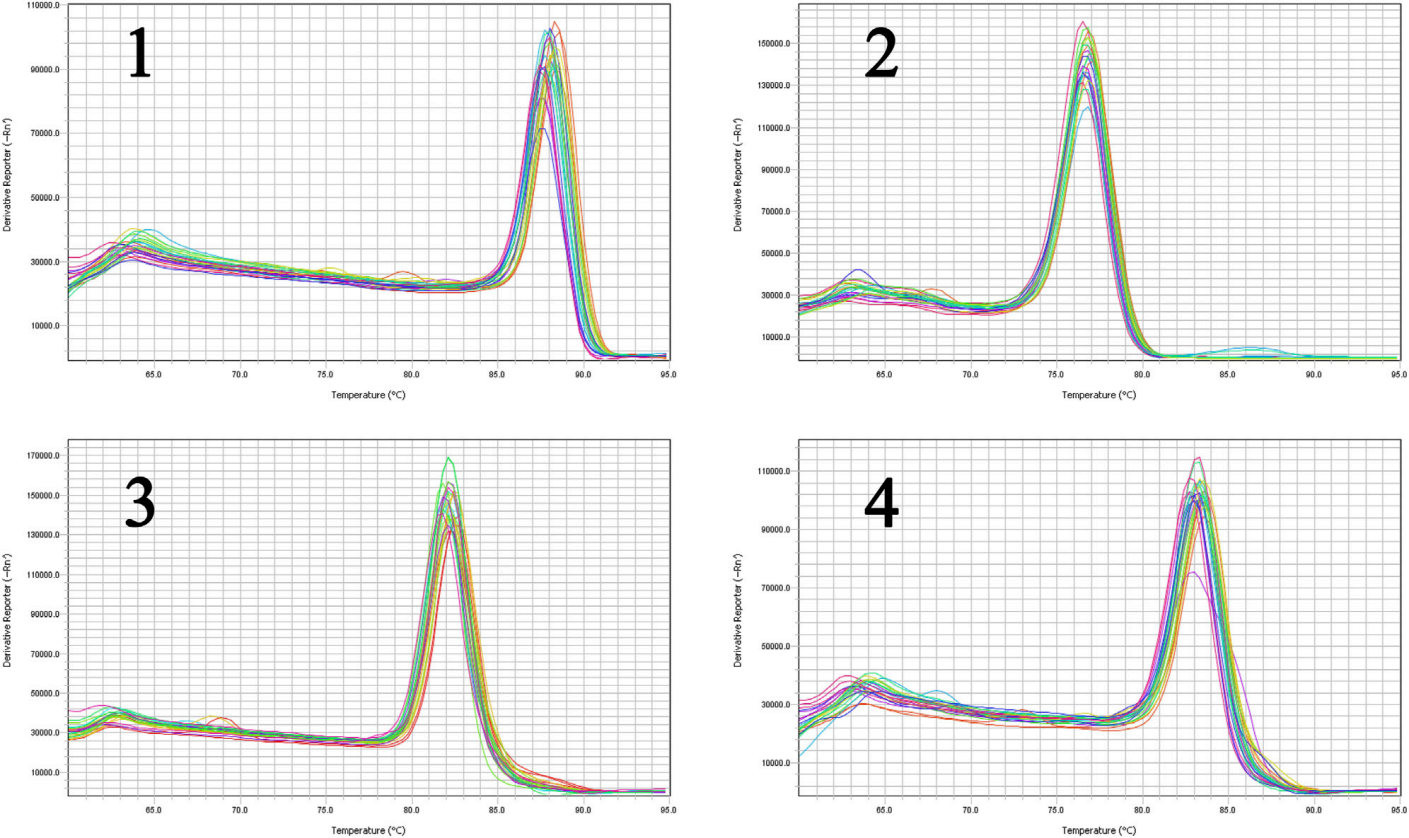
The melting curve of the four major genes (1, SREBP-2; 2, HMGCR; 3, MAPK1; 4, NF-*κ*B).

The liver tissues of blank, model and high-dose group rats were further conducted RT-qPCR. The results indicated that the gene expression of SREBP-2 and HMGCR of high-dose group rats decreased significantly (*p* < 0.05) in comparison with the blank group. The expression of MAPK1 and NF-κBp65 demonstrated an attenuation trend, which was however not significant (Figure 15). Figures 16, 17 show the amplification and melting curves of these four genes (Figures 16, 17).

## Discussion

### Identification of NAFLD-Related Targets of ZXD Treatment

ZXD is one of the TCMFs that exhibits anti-inflammatory and lipid-lowering properties. ZXD has been used to treat NAFLD because of hyperlipidemia- and inflammation-related etiology of this disease. However, the multi-component multi-target nature of ZXD and other TCMFs has made it difficult to firmly establish the molecular mechanisms underlying their pharmacological activity. In our previous study, we used LC-MS to conduct ZXD quality control analysis (Chang et al., 2021). This analysis, together with a review of relevant pharmacologically active compounds documented in the literature, revealed that alisol A (Ho et al., 2019), alisol A 24-acetate (Li et al., 2016), alisol A 23-acetate (Lin, 2012), 25-anhydroalisol F (Bi et al., 2017), alisol F (Bi et al., 2017), and alisol M 23-acetate (Xu et al., 2018) in ZX and atractylenolide I (Chao et al., 2016; Tang et al., 2017), atractylenolide II (Chao et al., 2016), and atractylenolide III (Song et al., 2017) in BZ are highly potent despite not meeting the predictive thresholds of the TCMSP platform (OB ≥ 30% and DL ≥ 0.18). As such, OB and DL alone are not sufficient to predict the active compounds within TCMFs.

The Swiss Target Platform was then used to identify putative NAFLD-related target genes. Further, we constructed a herb-compound-target network that identified HMGCR, MAPK1, and SREBP-2 as targets with high degree values that may be relevant in the context of ZXD-mediated NAFLD treatment.

### Targets Selected for Validation

HMGCR is the rate-limiting enzyme that regulates cholesterol synthesis. Patients with NAFLD are reported to have high serum levels of total cholesterol (TC) (Puri et al., 2007; Caballero et al., 2009). The gene expression of HMGCR, however, showed an abnormally elevated serum level of TC. The expression of HMGCR is regulated by SREBP, especially by SREBP-2, a transcription factor primarily involved in the synthesis of cholesterol and fatty acid (Brown and GOLDSTEIN, 1997; Wu et al., 2013). Therefore, we hypothesized that HMGCR and SREBP-2 induce the onset and development of NAFLD by affecting the serum level of TC. There are reports that ZXD suppresses the expression of HMGCR and SREBP-2 (Zheng and WU, 2010; Dan et al., 2011). Further, the major pharmacological components of ZXD - alisol B 23-acetate and alisol A 24-acetate - could bind to HMGCR competitively (Xu et al., 2016b).

MAPK signaling plays a central role in many physiological and pathological processes associated with inflammation and NAFLD (Cao et al., 2017; Jiang et al., 2019). MAPK1, MAPK8, and MAPK9 were high-scoring targets in our analysis and may thus be associated with NAFLD treatment. The pharmacologically active components of BZ are reported to modulate MAPK signaling (Huang et al., 2014). For instance, atractylenolide I inhibits MAPK by suppressing TLR4/MyD88 pathway activity, while atractylenolide II and III suppress inflammation by affecting MAPK phosphorylation.

PI3K/AKT/NF-*κ*B pathways were found to be closely associated with ZXD-mediated NAFLD treatment in PPI network analyses. Since many studies (Wang et al., 2014; Fan et al., 2017) have confirmed the relationship between NAFLD and the PI3K/AKT/NF-*κ*B pathway, NF-*κ*Bp65, the critical target of the PI3K/AKT/NF-*κ*B signaling pathway, was selected to validate our findings. Furthermore, HMGCR, MAPK1, SREBP-2, and NF-*κ*Bp65 were selected for further validation through RT-qPCR.

### RT-qPCR Experimental Validation Using *in vivo* NAFLD Model in SD Rats

The NAFLD model in rats was established using HFD and the success of modeling was verified by comparing changes in body weight and liver tissue section. The curative effect of ZXD was evaluated by histopathological evaluation and serum biochemical assays, and ZXD ameliorated lipid accumulation and treated NAFLD in a dose-dependent manner.

For validation purposes, RT-qPCR was performed to test the expression of HMGCR, MAPK1, SREBP-2, and NF-*κ*Bp65. ZXD significantly attenuated the expression of HMGCR and SREBP-2 (*p* < 0.05). The expression of MAPK1 and NF-κBp65 demonstrated an attenuation trend, which was however not significant (*p* > 0.05). The results indicated that our network pharmacology-based prediction model of molecular mechanism is reliable to some extent.

In future studies, we plan to perform a more in-depth investigation of the molecular mechanisms of ZXD based on the gut-liver axis to study the relationship between gut microbiota and NAFLD, using many novel databases of gut microbiota.

## Conclusion

In conclusion, we constructed a herb-compound-target-pathway network. Using biological enrichment analyses, we determined that ZXD can treat NAFLD in part by modulating the key targets - HMGCR, SREBP-2, MAPK1, and NF-*κ*Bp65. The experimental validation was performed through NAFLD model in SD rats. RT-qPCR analyses revealed that ZXD could significantly attenuated the expression of the four genes. The validation results indicated that our network pharmacology-based prediction model of molecular mechanism is reliable, which can be utilized for pilot mechanism investigation of TCMFs.

## Data Availability

The raw data supporting the conclusions of this article will be made available by the authors, without undue reservation, to any qualified researcher.
