# Supplementary figures and images for: Integrating Network Pharmacology and RT-qPCR Analysis to Investigate the Mechanisms Underlying ZeXie Decoction-Mediated Treatment of Non-alcoholic Fatty Liver Disease
Source: Front Pharmacol. 2021 Sep 9;12:722016. doi: 10.3389/fphar.2021.722016 (PMC8458890; doi:10.3389/fphar.2021.722016)

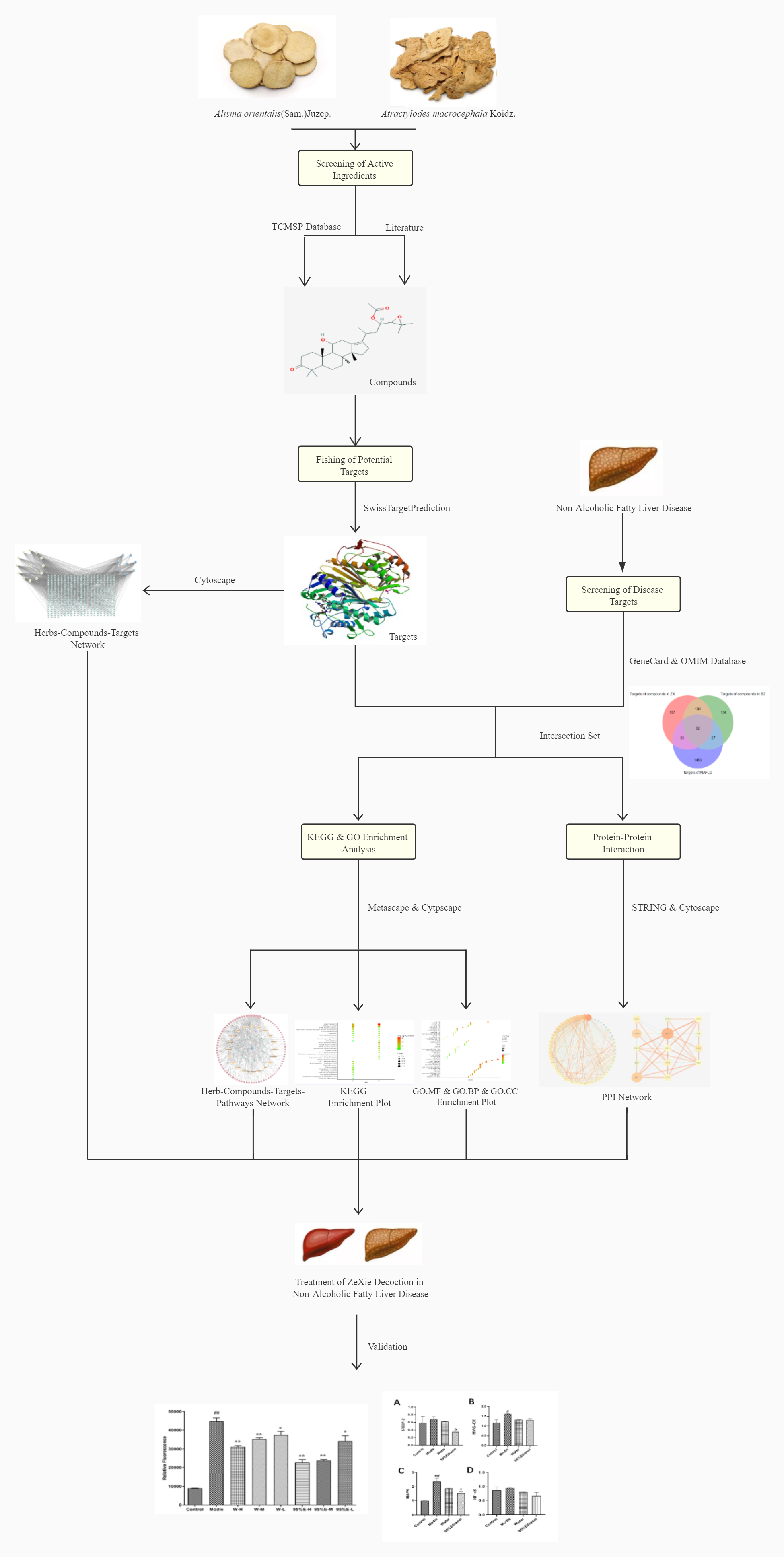

Supplement: Supplementary file 1 [file Image1.JPEG]
