# Supplementary material for: Integrating Network Pharmacology and RT-qPCR Analysis to Investigate the Mechanisms Underlying ZeXie Decoction-Mediated Treatment of Non-alcoholic Fatty Liver Disease
Source: Front Pharmacol. 2021 Sep 9;12:722016. doi: 10.3389/fphar.2021.722016 (PMC8458890; doi:10.3389/fphar.2021.722016)
Supplement: Supplementary file 2 [file DataSheet1.docx]

| **Table S1.** Information of targets predicted by Swiss platform | | | |
| --- | --- | --- | --- |
| Uniprot ID | Gene symbol | Protein name | Source |
| P08183 | ABCB1 | ATP-dependent translocase ABCB1 | ZX |
| P00519 | ABL1 | Tyrosine-protein kinase ABL1 | ZX |
| O00763 | ACACB | Acetyl-CoA carboxylase 2 | ZX |
| P25106 | ACKR3 | Atypical chemokine receptor 3 | ZX |
| P00813 | ADA | Adenosine deaminase | ZX |
| P29274 | ADORA2A | Adenosine receptor A2a | ZX |
| P29275 | ADORA2B | Adenosine receptor A2b | ZX |
| P35348 | ADRA1A | Alpha-1A adrenergic receptor | ZX |
| P35368 | ADRA1B | Alpha-1B adrenergic receptor | ZX |
| P25100 | ADRA1D | Alpha-1D adrenergic receptor | ZX |
| Q01433 | AMPD2 | AMP deaminase 2 | ZX |
| P05067 | APP | Amyloid-beta precursor protein | ZX |
| O14983 | ATP2A1 | Sarcoplasmic/endoplasmic reticulum calcium ATPase 1 | ZX |
| Q9NWT8 | AURKAIP1 | Aurora kinase A-interacting protein | ZX |
| P37288 | AVPR1A | Vasopressin V1a receptor | ZX |
| P30530 | AXL | Tyrosine-protein kinase receptor UFO | ZX |
| P32239 | CCKBR | Gastrin/cholecystokinin type B receptor | ZX |
| P20248 | CCNA2 | Cyclin-A2 | ZX |
| P24864 | CCNE1 | G1/S-specific cyclin-E1 | ZX |
| O60563 | CCNT1 | Cyclin-T1 | ZX |
| Q00526 | CDK3 | Cyclin-dependent kinase 3 | ZX |
| P49336 | CDK8 | Cyclin-dependent kinase 8 | ZX |
| P50750 | CDK9 | Cyclin-dependent kinase 9 | ZX |
| P11597 | CETP | Cholesteryl ester transfer protein | ZX |
| O14757 | CHEK1 | Serine/threonine-protein kinase Chk1 | ZX |
| O15111 | CHUK | Inhibitor of nuclear factor kappa-B kinase subunit alpha | ZX |
| P49759 | CLK1 | Dual specificity protein kinase CLK1 | ZX |
| P49760 | CLK2 | Dual specificity protein kinase CLK2 | ZX |
| Q9HAZ1 | CLK4 | Dual specificity protein kinase CLK4 | ZX |
| P34998 | CRHR1 | Corticotropin-releasing factor receptor 1 | ZX |
| P48730 | CSNK1D | Casein kinase I isoform delta | ZX |
| P07339 | CTSD | Cathepsin D | ZX |
| P25025 | CXCR2 | C-X-C chemokine receptor type 2 | ZX |
| P21728 | DRD1 | D(1A) dopamine receptor | ZX |
| P21917 | DRD4 | D(4) dopamine receptor | ZX |
| P33316 | DUT | Deoxyuridine 5'-triphosphate nucleotidohydrolase, mitochondrial | ZX |
| Q13627 | DYRK1A | Dual specificity tyrosine-phosphorylation-regulated kinase 1A | ZX |
| Q9Y463 | DYRK1B | Dual specificity tyrosine-phosphorylation-regulated kinase 1B | ZX |
| P24530 | EDNRB | Endothelin receptor type B | ZX |
| P08246 | ELANE | Neutrophil elastase | ZX |
| Q13822 | ENPP2 | Ectonucleotide pyrophosphatase/phosphodiesterase family member 2 | ZX |
| P54753 | EPHB3 | Ephrin type-B receptor 3 | ZX |
| P04626 | ERBB2 | Receptor tyrosine-protein kinase erbB-2 | ZX |
| Q15910 | EZH2 | Histone-lysine N-methyltransferase EZH2 | ZX |
| P00742 | F10 | Coagulation factor X | ZX |
| P00740 | F9 | Coagulation factor IX | ZX |
| P49327 | FASN | Fatty acid synthase | ZX |
| P62942 | FKBP1A | Peptidyl-prolyl cis-trans isomerase FKBP1A | ZX |
| P35557 | GCK | Hexokinase-4 | ZX |
| Q9GZN0 | GPR88 | Probable G-protein coupled receptor 88 | ZX |
| P42262 | GRIA2 | Glutamate receptor 2 | ZX |
| Q12879 | GRIN2A | Glutamate receptor ionotropic, NMDA 2A | ZX |
| P49841 | GSK3B | Glycogen synthase kinase-3 beta | ZX |
| P13807 | GYS1 | Glycogen [starch] synthase, muscle | ZX |
| Q9BY41 | HDAC8 | Histone deacetylase 8 | ZX |
| P37059 | HSD17B2 | Estradiol 17-beta-dehydrogenase 2 | ZX |
| P07900 | HSP90AA1 | Heat shock protein HSP 90-alpha | ZX |
| P08238 | HSP90AB1 | Heat shock protein HSP 90-beta | ZX |
| O75874 | IDH1 | Isocitrate dehydrogenase [NADP] cytoplasmic | ZX |
| P40189 | IL6ST | Interleukin-6 receptor subunit beta | ZX |
| P20839 | IMPDH1 | Inosine-5'-monophosphate dehydrogenase 1 | ZX |
| Q9NQS7 | INCENP | Inner centromere protein | ZX |
| P06213 | INSR | Insulin receptor | ZX |
| Q08881 | ITK | Tyrosine-protein kinase ITK/TSK | ZX |
| P22001 | KCNA3 | Potassium voltage-gated channel subfamily A member 3 | ZX |
| P10721 | KIT | Mast/stem cell growth factor receptor Kit | ZX |
| P53671 | LIMK2 | LIM domain kinase 2 | ZX |
| Q9H1C0 | LPAR5 | Lysophosphatidic acid receptor 5 | ZX |
| P43657 | LPAR6 | Lysophosphatidic acid receptor 6 | ZX |
| P11137 | MAP2 | Microtubule-associated protein 2 | ZX |
| Q99558 | MAP3K14 | Mitogen-activated protein kinase kinase kinase 14 | ZX |
| P53779 | MAPK10 | Mitogen-activated protein kinase 10 | ZX |
| Q15759 | MAPK11 | Mitogen-activated protein kinase 11 | ZX |
| O15264 | MAPK13 | Mitogen-activated protein kinase 13 | ZX |
| P45984 | MAPK9 | Mitogen-activated protein kinase 9 | ZX |
| O60307 | MAST3 | Microtubule-associated serine/threonine-protein kinase 3 | ZX |
| Q01726 | MC1R | Melanocyte-stimulating hormone receptor | ZX |
| P41968 | MC3R | Melanocortin receptor 3 | ZX |
| P32245 | MC4R | Melanocortin receptor 4 | ZX |
| P33032 | MC5R | Melanocortin receptor 5 | ZX |
| Q99705 | MCHR1 | Melanin-concentrating hormone receptor 1 | ZX |
| Q12866 | MERTK | Tyrosine-protein kinase Mer | ZX |
| P39900 | MMP12 | Macrophage metalloelastase | ZX |
| P08253 | MMP2 | 72 kDa type IV collagenase | ZX |
| P08254 | MMP3 | Stromelysin-1 | ZX |
| P09237 | MMP7 | Matrilysin | ZX |
| P42345 | MTOR | Serine/threonine-protein kinase mTOR | ZX |
| P43490 | NAMPT | Nicotinamide phosphoribosyltransferase | ZX |
| P30419 | NMT1 | Glycylpeptide N-tetradecanoyltransferase 1 | ZX |
| O75469 | NR1I2 | Nuclear receptor subfamily 1 group I member 2 | ZX |
| P04629 | NTRK1 | High affinity nerve growth factor receptor | ZX |
| P41143 | OPRD1 | Delta-type opioid receptor | ZX |
| P30559 | OXTR | Oxytocin receptor | ZX |
| P56373 | P2RX3 | P2X purinoceptor 3 | ZX |
| Q13153 | PAK1 | Serine/threonine-protein kinase PAK 1 | ZX |
| Q13370 | PDE3B | cGMP-inhibited 3',5'-cyclic phosphodiesterase B | ZX |
| P16234 | PDGFRA | Platelet-derived growth factor receptor alpha | ZX |
| Q15118 | PDK1 | [Pyruvate dehydrogenase | ZX |
| O15530 | PDPK1 | 3-phosphoinositide-dependent protein kinase 1 | ZX |
| O15055 | PER2 | Period circadian protein homolog 2 | ZX |
| Q16875 | PFKFB3 | 6-phosphofructo-2-kinase/fructose-2,6-bisphosphatase 3 | ZX |
| P0DJD9 | PGA5 | Pepsin A-5 | ZX |
| P53609 | PGGT1B | Geranylgeranyl transferase type-1 subunit beta | ZX |
| Q9UBF8 | PI4KB | Phosphatidylinositol 4-kinase beta | ZX |
| Q8NEB9 | PIK3C3 | Phosphatidylinositol 3-kinase catalytic subunit type 3 | ZX |
| P42338 | PIK3CB | Phosphatidylinositol 4,5-bisphosphate 3-kinase catalytic subunit beta isoform | ZX |
| P48736 | PIK3CG | Phosphatidylinositol 4,5-bisphosphate 3-kinase catalytic subunit gamma isoform | ZX |
| P19174 | PLCG1 | 1-phosphatidylinositol 4,5-bisphosphate phosphodiesterase gamma-1 | ZX |
| P09884 | POLA1 | DNA polymerase alpha catalytic subunit | ZX |
| O75688 | PPM1B | Protein phosphatase 1B | ZX |
| Q15172 | PPP2R5A | Serine/threonine-protein phosphatase 2A 56 kDa regulatory subunit alpha isoform | ZX |
| P05771 | PRKCB | Protein kinase C beta type | ZX |
| Q05655 | PRKCD | Protein kinase C delta type | ZX |
| Q02156 | PRKCE | Protein kinase C epsilon type | ZX |
| P05129 | PRKCG | Protein kinase C gamma type | ZX |
| Q04759 | PRKCQ | Protein kinase C theta type | ZX |
| Q05513 | PRKCZ | Protein kinase C zeta type | ZX |
| Q13882 | PTK6 | Protein-tyrosine kinase 6 | ZX |
| P11217 | PYGM | Glycogen phosphorylase, muscle form | ZX |
| P02753 | RBP4 | Retinol-binding protein 4 | ZX |
| P00797 | REN | Renin | ZX |
| O95977 | S1PR4 | Sphingosine 1-phosphate receptor 4 | ZX |
| Q9H228 | S1PR5 | Sphingosine 1-phosphate receptor 5 | ZX |
| P13866 | SLC5A1 | Sodium/glucose cotransporter 1 | ZX |
| P32418 | SLC8A1 | Sodium/calcium exchanger 1 | ZX |
| P35610 | SOAT1 | Sterol O-acyltransferase 1 | ZX |
| O75908 | SOAT2 | Sterol O-acyltransferase 2 | ZX |
| Q00796 | SORD | Sorbitol dehydrogenase | ZX |
| P43405 | SYK | Tyrosine-protein kinase SYK | ZX |
| P21452 | TACR2 | Substance-K receptor | ZX |
| P04183 | TK1 | Thymidine kinase, cytosolic | ZX |
| O00142 | TK2 | Thymidine kinase 2, mitochondrial | ZX |
| Q12931 | TRAP1 | Heat shock protein 75 kDa, mitochondrial | ZX |
| Q8NER1 | TRPV1 | Transient receptor potential cation channel subfamily V member 1 | ZX |
| Q9HBA0 | TRPV4 | Transient receptor potential cation channel subfamily V member 4 | ZX |
| P29597 | TYK2 | Non-receptor tyrosine-protein kinase TYK2 | ZX |
| Q06418 | TYRO3 | Tyrosine-protein kinase receptor TYRO3 | ZX |
| P15498 | VAV1 | Proto-oncogene vav | ZX |
| P40337 | VHL | von Hippel-Lindau disease tumor suppressor | ZX |
| P22303 | ACHE | Acetylcholinesterase | ZX and BZ |
| P0DMS8 | ADORA3 | Adenosine receptor A3 | ZX and BZ |
| O60218 | AKR1B10 | Aldo-keto reductase family 1 member B10 | ZX and BZ |
| Q9UM73 | ALK | ALK tyrosine kinase receptor | ZX and BZ |
| P09917 | ALOX5 | Arachidonate 5-lipoxygenase | ZX and BZ |
| P10275 | AR | Androgen receptor | ZX and BZ |
| O14965 | AURKA | Aurora kinase A | ZX and BZ |
| Q96GD4 | AURKB | Aurora kinase B | ZX and BZ |
| P30518 | AVPR2 | Vasopressin V2 receptor | ZX and BZ |
| P56817 | BACE1 | Beta-secretase 1 | ZX and BZ |
| Q9Y5Z0 | BACE2 | Beta-secretase 2 | ZX and BZ |
| P06276 | BCHE | Cholinesterase | ZX and BZ |
| P21730 | C5AR1 | C5a anaphylatoxin chemotactic receptor 1 | ZX and BZ |
| P41180 | CASR | Extracellular calcium-sensing receptor | ZX and BZ |
| Q8WWL7 | CCNB3 | G2/mitotic-specific cyclin-B3 | ZX and BZ |
| P24863 | CCNC | Cyclin-C | ZX and BZ |
| P24385 | CCND1 | G1/S-specific cyclin-D1 | ZX and BZ |
| O96020 | CCNE2 | G1/S-specific cyclin-E2 | ZX and BZ |
| P32246 | CCR1 | C-C chemokine receptor type 1 | ZX and BZ |
| P60033 | CD81 | CD81 antigen | ZX and BZ |
| P30305 | CDC25B | M-phase inducer phosphatase 2 | ZX and BZ |
| P06493 | CDK1 | Cyclin-dependent kinase 1 | ZX and BZ |
| P24941 | CDK2 | Cyclin-dependent kinase 2 | ZX and BZ |
| P11802 | CDK4 | Cyclin-dependent kinase 4 | ZX and BZ |
| Q00535 | CDK5 | Cyclin-dependent-like kinase 5 | ZX and BZ |
| Q00534 | CDK6 | Cyclin-dependent kinase 6 | ZX and BZ |
| O00748 | CES2 | Cocaine esterase | ZX and BZ |
| P00746 | CFD | Complement factor D | ZX and BZ |
| P08172 | CHRM2 | Muscarinic acetylcholine receptor M2 | ZX and BZ |
| P21554 | CNR1 | Cannabinoid receptor 1 | ZX and BZ |
| P34972 | CNR2 | Cannabinoid receptor 2 | ZX and BZ |
| P07333 | CSF1R | Macrophage colony-stimulating factor 1 receptor | ZX and BZ |
| P43235 | CTSK | Cathepsin K | ZX and BZ |
| P07711 | CTSL | Cathepsin L1 | ZX and BZ |
| P25774 | CTSS | Cathepsin S | ZX and BZ |
| P25024 | CXCR1 | C-X-C chemokine receptor type 1 | ZX and BZ |
| P15538 | CYP11B1 | Cytochrome P450 11B1, mitochondrial | ZX and BZ |
| P19099 | CYP11B2 | Cytochrome P450 11B2, mitochondrial | ZX and BZ |
| P05093 | CYP17A1 | Steroid 17-alpha-hydroxylase/17,20 lyase | ZX and BZ |
| P11511 | CYP19A1 | Aromatase | ZX and BZ |
| P33261 | CYP2C19 | Cytochrome P450 2C19 | ZX and BZ |
| Q16850 | CYP51A1 | Lanosterol 14-alpha demethylase | ZX and BZ |
| Q9UBM7 | DHCR7 | 7-dehydrocholesterol reductase | ZX and BZ |
| P35462 | DRD3 | D(3) dopamine receptor | ZX and BZ |
| P00533 | EGFR | Epidermal growth factor receptor | ZX and BZ |
| P54760 | EPHB4 | Ephrin type-B receptor 4 | ZX and BZ |
| P34913 | EPHX2 | Bifunctional epoxide hydrolase 2 [Includes: Cytosolic epoxide hydrolase 2 | ZX and BZ |
| P03372 | ESR1 | Estrogen receptor | ZX and BZ |
| Q92731 | ESR2 | Estrogen receptor beta | ZX and BZ |
| P00734 | F2 | Prothrombin | ZX and BZ |
| P25116 | F2R | Proteinase-activated receptor 1 | ZX and BZ |
| P55085 | F2RL1 | Proteinase-activated receptor 2 | ZX and BZ |
| O00519 | FAAH | Fatty-acid amide hydrolase 1 | ZX and BZ |
| P05413 | FABP3 | Fatty acid-binding protein, heart | ZX and BZ |
| Q01469 | FABP5 | Fatty acid-binding protein 5 | ZX and BZ |
| Q12884 | FAP | Prolyl endopeptidase FAP | ZX and BZ |
| P37268 | FDFT1 | Squalene synthase | ZX and BZ |
| P36888 | FLT3 | Receptor-type tyrosine-protein kinase FLT3 | ZX and BZ |
| P35916 | FLT4 | Vascular endothelial growth factor receptor 3 | ZX and BZ |
| P49354 | FNTA | Protein farnesyltransferase/geranylgeranyltransferase type-1 subunit alpha | ZX and BZ |
| P06241 | FYN | Tyrosine-protein kinase Fyn | ZX and BZ |
| P11413 | G6PD | Glucose-6-phosphate 1-dehydrogenase | ZX and BZ |
| P47869 | GABRA2 | Gamma-aminobutyric acid receptor subunit alpha-2 | ZX and BZ |
| P28472 | GABRB3 | Gamma-aminobutyric acid receptor subunit beta-3 | ZX and BZ |
| P23415 | GLRA1 | Glycine receptor subunit alpha-1 | ZX and BZ |
| P49840 | GSK3A | Glycogen synthase kinase-3 alpha | ZX and BZ |
| O43613 | HCRTR1 | Orexin receptor type 1 | ZX and BZ |
| O43614 | HCRTR2 | Orexin receptor type 2 | ZX and BZ |
| P04035 | HMGCR | 3-hydroxy-3-methylglutaryl-coenzyme A reductase | ZX and BZ |
| P28845 | HSD11B1 | Corticosteroid 11-beta-dehydrogenase isozyme 1 | ZX and BZ |
| P80365 | HSD11B2 | Corticosteroid 11-beta-dehydrogenase isozyme 2 | ZX and BZ |
| P37058 | HSD17B3 | Testosterone 17-beta-dehydrogenase 3 | ZX and BZ |
| P28223 | HTR2A | 5-hydroxytryptamine receptor 2A | ZX and BZ |
| P28335 | HTR2C | 5-hydroxytryptamine receptor 2C | ZX and BZ |
| P41252 | IARS | Isoleucine--tRNA ligase, cytoplasmic | ZX and BZ |
| P14902 | IDO1 | Indoleamine 2,3-dioxygenase 1 | ZX and BZ |
| P08069 | IGF1R | Insulin-like growth factor 1 receptor | ZX and BZ |
| O14920 | IKBKB | Inhibitor of nuclear factor kappa-B kinase subunit beta | ZX and BZ |
| P12268 | IMPDH2 | Inosine-5'-monophosphate dehydrogenase 2 | ZX and BZ |
| Q9NWZ3 | IRAK4 | Interleukin-1 receptor-associated kinase 4 | ZX and BZ |
| P20701 | ITGAL | Integrin alpha-L | ZX and BZ |
| P23458 | JAK1 | Tyrosine-protein kinase JAK1 | ZX and BZ |
| O60674 | JAK2 | Tyrosine-protein kinase JAK2 | ZX and BZ |
| P52333 | JAK3 | Tyrosine-protein kinase JAK3 | ZX and BZ |
| P05412 | JUN | Transcription factor AP-1 | ZX and BZ |
| P22460 | KCNA5 | Potassium voltage-gated channel subfamily A member 5 | ZX and BZ |
| Q12809 | KCNH2 | Potassium voltage-gated channel subfamily H member 2 | ZX and BZ |
| O14649 | KCNK3 | Potassium channel subfamily K member 3 | ZX and BZ |
| P35968 | KDR | Vascular endothelial growth factor receptor 2 | ZX and BZ |
| P52732 | KIF11 | Kinesin-like protein KIF11 | ZX and BZ |
| Q5S007 | LRRK2 | Leucine-rich repeat serine/threonine-protein kinase 2 | ZX and BZ |
| Q15722 | LTB4R | Leukotriene B4 receptor 1 | ZX and BZ |
| Q02750 | MAP2K1 | Dual specificity mitogen-activated protein kinase kinase 1 | ZX and BZ |
| P28482 | MAPK1 | Mitogen-activated protein kinase 1 | ZX and BZ |
| Q16539 | MAPK14 | Mitogen-activated protein kinase 14 | ZX and BZ |
| P27361 | MAPK3 | Mitogen-activated protein kinase 3 | ZX and BZ |
| P45983 | MAPK8 | Mitogen-activated protein kinase 8 | ZX and BZ |
| P49137 | MAPKAPK2 | MAP kinase-activated protein kinase 2 | ZX and BZ |
| Q00987 | MDM2 | E3 ubiquitin-protein ligase Mdm2 | ZX and BZ |
| O15151 | MDM4 | Protein Mdm4 | ZX and BZ |
| P08581 | MET | Hepatocyte growth factor receptor | ZX and BZ |
| P50579 | METAP2 | Methionine aminopeptidase 2 | ZX and BZ |
| P03956 | MMP1 | Interstitial collagenase | ZX and BZ |
| P48039 | MTNR1A | Melatonin receptor type 1A | ZX and BZ |
| P49286 | MTNR1B | Melatonin receptor type 1B | ZX and BZ |
| P35228 | NOS2 | Nitric oxide synthase, inducible | ZX and BZ |
| Q9UHC9 | NPC1L1 | NPC1-like intracellular cholesterol transporter 1 | ZX and BZ |
| Q15761 | NPY5R | Neuropeptide Y receptor type 5 | ZX and BZ |
| P55055 | NR1H2 | Oxysterols receptor LXR-beta | ZX and BZ |
| Q13133 | NR1H3 | Oxysterols receptor LXR-alpha | ZX and BZ |
| Q96RI1 | NR1H4 | Bile acid receptor | ZX and BZ |
| Q14994 | NR1I3 | Nuclear receptor subfamily 1 group I member 3 | ZX and BZ |
| P04150 | NR3C1 | Glucocorticoid receptor | ZX and BZ |
| P08235 | NR3C2 | Mineralocorticoid receptor | ZX and BZ |
| P35372 | OPRM1 | Mu-type opioid receptor | ZX and BZ |
| Q99572 | P2RX7 | P2X purinoceptor 7 | ZX and BZ |
| P09874 | PARP1 | Poly [ADP-ribose] polymerase 1 | ZX and BZ |
| Q53EL6 | PDCD4 | Programmed cell death protein 4 | ZX and BZ |
| Q9Y233 | PDE10A | cAMP and cAMP-inhibited cGMP 3',5'-cyclic phosphodiesterase 10A | ZX and BZ |
| O00408 | PDE2A | cGMP-dependent 3',5'-cyclic phosphodiesterase | ZX and BZ |
| Q14432 | PDE3A | cGMP-inhibited 3',5'-cyclic phosphodiesterase A | ZX and BZ |
| Q07343 | PDE4B | cAMP-specific 3',5'-cyclic phosphodiesterase 4B | ZX and BZ |
| Q08499 | PDE4D | cAMP-specific 3',5'-cyclic phosphodiesterase 4D | ZX and BZ |
| P09619 | PDGFRB | Platelet-derived growth factor receptor beta | ZX and BZ |
| P06401 | PGR | Progesterone receptor | ZX and BZ |
| P42336 | PIK3CA | Phosphatidylinositol 4,5-bisphosphate 3-kinase catalytic subunit alpha isoform | ZX and BZ |
| O00329 | PIK3CD | Phosphatidylinositol 4,5-bisphosphate 3-kinase catalytic subunit delta isoform | ZX and BZ |
| P11309 | PIM1 | Serine/threonine-protein kinase pim-1 | ZX and BZ |
| Q86V86 | PIM3 | Serine/threonine-protein kinase pim-3 | ZX and BZ |
| P04054 | PLA2G1B | Phospholipase A2 | ZX and BZ |
| P14555 | PLA2G2A | Phospholipase A2, membrane associated | ZX and BZ |
| P06746 | POLB | DNA polymerase beta | ZX and BZ |
| Q07869 | PPARA | Peroxisome proliferator-activated receptor alpha | ZX and BZ |
| Q03181 | PPARD | Peroxisome proliferator-activated receptor delta | ZX and BZ |
| P37231 | PPARG | Peroxisome proliferator-activated receptor gamma | ZX and BZ |
| P36873 | PPP1CC | Serine/threonine-protein phosphatase PP1-gamma catalytic subunit | ZX and BZ |
| P67775 | PPP2CA | Serine/threonine-protein phosphatase 2A catalytic subunit alpha isoform | ZX and BZ |
| P48147 | PREP | Prolyl endopeptidase | ZX and BZ |
| P17252 | PRKCA | Protein kinase C alpha type | ZX and BZ |
| P24723 | PRKCH | Protein kinase C eta type | ZX and BZ |
| P49810 | PSEN2 | Presenilin-2 | ZX and BZ |
| P25105 | PTAFR | Platelet-activating factor receptor | ZX and BZ |
| Q13258 | PTGDR | Prostaglandin D2 receptor | ZX and BZ |
| P34995 | PTGER1 | Prostaglandin E2 receptor EP1 subtype | ZX and BZ |
| P43116 | PTGER2 | Prostaglandin E2 receptor EP2 subtype | ZX and BZ |
| P35408 | PTGER4 | Prostaglandin E2 receptor EP4 subtype | ZX and BZ |
| O14684 | PTGES | Prostaglandin E synthase | ZX and BZ |
| P23219 | PTGS1 | Prostaglandin G/H synthase 1 | ZX and BZ |
| P35354 | PTGS2 | Prostaglandin G/H synthase 2 | ZX and BZ |
| P18031 | PTPN1 | Tyrosine-protein phosphatase non-receptor type 1 | ZX and BZ |
| Q06124 | PTPN11 | Tyrosine-protein phosphatase non-receptor type 11 | ZX and BZ |
| P17706 | PTPN2 | Tyrosine-protein phosphatase non-receptor type 2 | ZX and BZ |
| P29350 | PTPN6 | Tyrosine-protein phosphatase non-receptor type 6 | ZX and BZ |
| P06737 | PYGL | Glycogen phosphorylase, liver form | ZX and BZ |
| Q13464 | ROCK1 | Rho-associated protein kinase 1 | ZX and BZ |
| O75116 | ROCK2 | Rho-associated protein kinase 2 | ZX and BZ |
| P35398 | RORA | Nuclear receptor ROR-alpha | ZX and BZ |
| P51449 | RORC | Nuclear receptor ROR-gamma | ZX and BZ |
| P23443 | RPS6KB1 | Ribosomal protein S6 kinase beta-1 | ZX and BZ |
| P21453 | S1PR1 | Sphingosine 1-phosphate receptor 1 | ZX and BZ |
| Q99500 | S1PR3 | Sphingosine 1-phosphate receptor 3 | ZX and BZ |
| O00767 | SCD | Acyl-CoA desaturase | ZX and BZ |
| Q14524 | SCN5A | Sodium channel protein type 5 subunit alpha | ZX and BZ |
| Q15858 | SCN9A | Sodium channel protein type 9 subunit alpha | ZX and BZ |
| P08185 | SERPINA6 | Corticosteroid-binding globulin | ZX and BZ |
| P04278 | SHBG | Sex hormone-binding globulin | ZX and BZ |
| Q99720 | SIGMAR1 | Sigma non-opioid intracellular receptor 1 | ZX and BZ |
| Q8IXJ6 | SIRT2 | NAD-dependent protein deacetylase sirtuin-2 | ZX and BZ |
| P23975 | SLC6A2 | Sodium-dependent noradrenaline transporter | ZX and BZ |
| Q01959 | SLC6A3 | Sodium-dependent dopamine transporter | ZX and BZ |
| P31645 | SLC6A4 | Sodium-dependent serotonin transporter | ZX and BZ |
| Q99835 | SMO | Smoothened homolog | ZX and BZ |
| Q14534 | SQLE | Squalene monooxygenase | ZX and BZ |
| P12931 | SRC | Proto-oncogene tyrosine-protein kinase Src | ZX and BZ |
| P18405 | SRD5A1 | 3-oxo-5-alpha-steroid 4-dehydrogenase 1 | ZX and BZ |
| Q12772 | SREBF2 | Sterol regulatory element-binding protein 2 | ZX and BZ |
| P08842 | STS | Steryl-sulfatase | ZX and BZ |
| P25103 | TACR1 | Substance-P receptor | ZX and BZ |
| P21731 | TBXA2R | Thromboxane A2 receptor | ZX and BZ |
| O14746 | TERT | Telomerase reverse transcriptase | ZX and BZ |
| O95271 | TNKS | Poly [ADP-ribose] polymerase tankyrase-1 | ZX and BZ |
| Q9H2K2 | TNKS2 | Poly [ADP-ribose] polymerase tankyrase-2 | ZX and BZ |
| P33981 | TTK | Dual specificity protein kinase TTK | ZX and BZ |
| Q8NG68 | TTL | Tubulin--tyrosine ligase | ZX and BZ |
| P16662 | UGT2B7 | UDP-glucuronosyltransferase 2B7 | ZX and BZ |
| P11473 | VDR | Vitamin D3 receptor | ZX and BZ |
| P12821 | ACE | Angiotensin-converting enzyme | BZ |
| P24666 | ACP1 | Low molecular weight phosphotyrosine protein phosphatase | BZ |
| P07327 | ADH1A | Alcohol dehydrogenase 1A | BZ |
| P00326 | ADH1C | Alcohol dehydrogenase 1C | BZ |
| P08913 | ADRA2A | Alpha-2A adrenergic receptor | BZ |
| P18089 | ADRA2B | Alpha-2B adrenergic receptor | BZ |
| P18825 | ADRA2C | Alpha-2C adrenergic receptor | BZ |
| P30556 | AGTR1 | Type-1 angiotensin II receptor | BZ |
| P35869 | AHR | Aryl hydrocarbon receptor | BZ |
| P18054 | ALOX12 | Arachidonate 12-lipoxygenase, 12S-type | BZ |
| P16050 | ALOX15 | Arachidonate 15-lipoxygenase | BZ |
| P20292 | ALOX5AP | Arachidonate 5-lipoxygenase-activating protein | BZ |
| P10275 | AR | Androgen receptor | BZ |
| P54707 | ATP12A | Potassium-transporting ATPase alpha chain 2 | BZ |
| P05023 | ATP1A1 | Sodium/potassium-transporting ATPase subunit alpha-1 | BZ |
| P30518 | AVPR2 | Vasopressin V2 receptor | BZ |
| Q07817 | BCL2L1 | Bcl-2-like protein 1 | BZ |
| O43570 | CA12 | Carbonic anhydrase 12 | BZ |
| Q8N1Q1 | CA13 | Carbonic anhydrase 13 | BZ |
| Q9ULX7 | CA14 | Carbonic anhydrase 14 | BZ |
| P00918 | CA2 | Carbonic anhydrase 2 | BZ |
| P07451 | CA3 | Carbonic anhydrase 3 | BZ |
| P35218 | CA5A | Carbonic anhydrase 5A, mitochondrial | BZ |
| Q9Y2D0 | CA5B | Carbonic anhydrase 5B, mitochondrial | BZ |
| P23280 | CA6 | Carbonic anhydrase 6 | BZ |
| P43166 | CA7 | Carbonic anhydrase 7 | BZ |
| Q16790 | CA9 | Carbonic anhydrase 9 | BZ |
| P24863 | CCNC | Cyclin-C | BZ |
| P32246 | CCR1 | C-C chemokine receptor type 1 | BZ |
| P30304 | CDC25A | M-phase inducer phosphatase 1 | BZ |
| P30307 | CDC25C | M-phase inducer phosphatase 3 | BZ |
| P11802 | CDK4 | Cyclin-dependent kinase 4 | BZ |
| Q00534 | CDK6 | Cyclin-dependent kinase 6 | BZ |
| P19835 | CEL | Bile salt-activated lipase | BZ |
| P23141 | CES1 | Liver carboxylesterase 1 | BZ |
| P11229 | CHRM1 | Muscarinic acetylcholine receptor M1 | BZ |
| P20309 | CHRM3 | Muscarinic acetylcholine receptor M3 | BZ |
| P08173 | CHRM4 | Muscarinic acetylcholine receptor M4 | BZ |
| P08912 | CHRM5 | Muscarinic acetylcholine receptor M5 | BZ |
| P43681 | CHRNA4 | Neuronal acetylcholine receptor subunit alpha-4 | BZ |
| P07333 | CSF1R | Macrophage colony-stimulating factor 1 receptor | BZ |
| Q9HCP0 | CSNK1G1 | Casein kinase I isoform gamma-1 | BZ |
| P17538 | CTRB1 | Chymotrypsinogen B | BZ |
| P07858 | CTSB | Cathepsin B | BZ |
| P09668 | CTSH | Pro-cathepsin H [Cleaved into: Cathepsin H mini chain; Cathepsin H | BZ |
| P49682 | CXCR3 | C-X-C chemokine receptor type 3 | BZ |
| P11511 | CYP19A1 | Aromatase | BZ |
| P05177 | CYP1A2 | Cytochrome P450 1A2 | BZ |
| P11509 | CYP2A6 | Cytochrome P450 2A6 | BZ |
| P11712 | CYP2C9 | Cytochrome P450 2C9 | BZ |
| P10635 | CYP2D6 | Cytochrome P450 2D6 | BZ |
| P08684 | CYP3A4 | Cytochrome P450 3A4 | BZ |
| Q9H773 | DCTPP1 | dCTP pyrophosphatase 1 | BZ |
| Q02127 | DHODH | Dihydroorotate dehydrogenase | BZ |
| P14416 | DRD2 | D(2) dopamine receptor | BZ |
| Q9BQI3 | EIF2AK1 | Eukaryotic translation initiation factor 2-alpha kinase 1 | BZ |
| P07099 | EPHX1 | Epoxide hydrolase 1 | BZ |
| P34913 | EPHX2 | Bifunctional epoxide hydrolase 2 [Includes: Cytosolic epoxide hydrolase 2 | BZ |
| Q92731 | ESR2 | Estrogen receptor beta | BZ |
| P07148 | FABP1 | Fatty acid-binding protein, liver | BZ |
| P15090 | FABP4 | Fatty acid-binding protein, adipocyte | BZ |
| O60427 | FADS1 | Acyl-CoA | BZ |
| O14842 | FFAR1 | Free fatty acid receptor 1 | BZ |
| P47871 | GCGR | Glucagon receptor | BZ |
| Q9Y2T6 | GPR55 | G-protein coupled receptor 55 | BZ |
| Q14416 | GRM2 | Metabotropic glutamate receptor 2 | BZ |
| Q14833 | GRM4 | Metabotropic glutamate receptor 4 | BZ |
| P41594 | GRM5 | Metabotropic glutamate receptor 5 | BZ |
| O43613 | HCRTR1 | Orexin receptor type 1 | BZ |
| O43614 | HCRTR2 | Orexin receptor type 2 | BZ |
| Q16665 | HIF1A | Hypoxia-inducible factor 1-alpha | BZ |
| P09601 | HMOX1 | Heme oxygenase 1 | BZ |
| Q9Y5N1 | HRH3 | Histamine H3 receptor | BZ |
| Q9H3N8 | HRH4 | Histamine H4 receptor | BZ |
| P41595 | HTR2B | 5-hydroxytryptamine receptor 2B | BZ |
| P01584 | IL1B | Interleukin-1 beta | BZ |
| P20701 | ITGAL | Integrin alpha-L | BZ |
| Q12809 | KCNH2 | Potassium voltage-gated channel subfamily H member 2 | BZ |
| P48544 | KCNJ5 | G protein-activated inward rectifier potassium channel 4 | BZ |
| O95069 | KCNK2 | Potassium channel subfamily K member 2 | BZ |
| P06239 | LCK | Tyrosine-protein kinase Lck | BZ |
| P21397 | MAOA | Amine oxidase [flavin-containing] A | BZ |
| P27338 | MAOB | Amine oxidase [flavin-containing] B | BZ |
| Q02750 | MAP2K1 | Dual specificity mitogen-activated protein kinase kinase 1 | BZ |
| Q16539 | MAPK14 | Mitogen-activated protein kinase 14 | BZ |
| O15151 | MDM4 | Protein Mdm4 | BZ |
| Q99685 | MGLL | Monoglyceride lipase | BZ |
| P45452 | MMP13 | Collagenase 3 | BZ |
| P05164 | MPO | Myeloperoxidase | BZ |
| P00403 | MT-CO2 | Cytochrome c oxidase subunit 2 | BZ |
| P48039 | MTNR1A | Melatonin receptor type 1A | BZ |
| P49286 | MTNR1B | Melatonin receptor type 1B | BZ |
| P29475 | NOS1 | Nitric oxide synthase, brain | BZ |
| P29474 | NOS3 | Nitric oxide synthase, endothelial | BZ |
| P16083 | NQO2 | Ribosyldihydronicotinamide dehydrogenase [quinone] | BZ |
| P08235 | NR3C2 | Mineralocorticoid receptor | BZ |
| P35372 | OPRM1 | Mu-type opioid receptor | BZ |
| P11940 | PABPC1 | Polyadenylate-binding protein 1 | BZ |
| Q16549 | PCSK7 | Proprotein convertase subtilisin/kexin type 7 | BZ |
| P27815 | PDE4A | cAMP-specific 3',5'-cyclic phosphodiesterase 4A | BZ |
| Q08493 | PDE4C | cAMP-specific 3',5'-cyclic phosphodiesterase 4C | BZ |
| Q08499 | PDE4D | cAMP-specific 3',5'-cyclic phosphodiesterase 4D | BZ |
| O76074 | PDE5A | cGMP-specific 3',5'-cyclic phosphodiesterase | BZ |
| Q13946 | PDE7A | High affinity cAMP-specific 3',5'-cyclic phosphodiesterase 7A | BZ |
| O76083 | PDE9A | High affinity cGMP-specific 3',5'-cyclic phosphodiesterase 9A | BZ |
| P06401 | PGR | Progesterone receptor | BZ |
| P11309 | PIM1 | Serine/threonine-protein kinase pim-1 | BZ |
| Q86V86 | PIM3 | Serine/threonine-protein kinase pim-3 | BZ |
| P04054 | PLA2G1B | Phospholipase A2 | BZ |
| Q07869 | PPARA | Peroxisome proliferator-activated receptor alpha | BZ |
| P78527 | PRKDC | DNA-dependent protein kinase catalytic subunit | BZ |
| P49810 | PSEN2 | Presenilin-2 | BZ |
| Q13258 | PTGDR | Prostaglandin D2 receptor | BZ |
| P43119 | PTGIR | Prostacyclin receptor | BZ |
| Q05397 | PTK2 | Focal adhesion kinase 1 | BZ |
| Q14289 | PTK2B | Protein-tyrosine kinase 2-beta | BZ |
| P08575 | PTPRC | Receptor-type tyrosine-protein phosphatase C | BZ |
| P10586 | PTPRF | Receptor-type tyrosine-protein phosphatase F | BZ |
| Q16769 | QPCT | Glutaminyl-peptide cyclotransferase | BZ |
| Q8IV61 | RASGRP3 | Ras guanyl-releasing protein 3 | BZ |
| O43353 | RIPK2 | Receptor-interacting serine/threonine-protein kinase 2 | BZ |
| Q9Y572 | RIPK3 | Receptor-interacting serine/threonine-protein kinase 3 | BZ |
| Q15418 | RPS6KA1 | Ribosomal protein S6 kinase alpha-1 | BZ |
| O75582 | RPS6KA5 | Ribosomal protein S6 kinase alpha-5 | BZ |
| Q15393 | SF3B3 | Splicing factor 3B subunit 3 | BZ |
| Q6P1M0 | SLC27A4 | Long-chain fatty acid transport protein 4 | BZ |
| Q01959 | SLC6A3 | Sodium-dependent dopamine transporter | BZ |
| P40763 | STAT3 | Signal transducer and activator of transcription 3 | BZ |
| P59538 | TAS2R31 | Taste receptor type 2 member 31 | BZ |
| P24557 | TBXAS1 | Thromboxane-A synthase | BZ |
| P11387 | TOP1 | DNA topoisomerase 1 | BZ |
| P11388 | TOP2A | DNA topoisomerase 2-alpha | BZ |
| P07202 | TPO | Thyroid peroxidase | BZ |
| Q8NET8 | TRPV3 | Transient receptor potential cation channel subfamily V member 3 | BZ |
| P33981 | TTK | Dual specificity protein kinase TTK | BZ |
| Q8NG68 | TTL | Tubulin--tyrosine ligase | BZ |
| P02766 | TTR | Transthyretin | BZ |
| Q9H4B7 | TUBB1 | Tubulin beta-1 chain | BZ |
| P04818 | TYMS | Thymidylate synthase | BZ |
| Q9UKP6 | UTS2R | Urotensin-2 receptor | BZ |
| O14980 | XPO1 | Exportin-1 | BZ |

The protein names were retrieved in Uniprot database

| **Table S2.** Information of matched targets of ZXD and NAFLD | | |
| --- | --- | --- |
| Uniprot ID | Gene Symbol | Protein name |
| P08183 | ABCB1 | ATP-dependent translocase ABCB1 |
| O00763 | ACACB | Acetyl-CoA carboxylase 2 |
| P12821 | ACE | Angiotensin-converting enzyme |
| P22303 | ACHE | Acetylcholinesterase |
| P00326 | ADH1C | Alcohol dehydrogenase 1C |
| P30556 | AGTR1 | Type-1 angiotensin II receptor |
| P31749 | AKT1 | RAC-alpha serine/threonine-protein kinase |
| Q9UM73 | ALK | ALK tyrosine kinase receptor |
| P18054 | ALOX12 | Arachidonate 12-lipoxygenase, 12S-type |
| P05186 | ALPL | Alkaline phosphatase, tissue-nonspecific isozyme |
| P05067 | APP | Amyloid-beta precursor protein |
| P05023 | ATP1A1 | Sodium/potassium-transporting ATPase subunit alpha-1 |
| P24385 | CCND1 | G1/S-specific cyclin-D1 |
| P19835 | CEL | Bile salt-activated lipase |
| P23141 | CES1 | Liver carboxylesterase 1 |
| O00748 | CES2 | Cocaine esterase |
| P11597 | CETP | Cholesteryl ester transfer protein |
| O15111 | CHUK | Inhibitor of nuclear factor kappa-B kinase subunit alpha |
| P21554 | CNR1 | Cannabinoid receptor 1 |
| P34972 | CNR2 | Cannabinoid receptor 2 |
| P07858 | CTSB | Cathepsin B |
| P25774 | CTSS | Cathepsin S |
| P25025 | CXCR2 | C-X-C chemokine receptor type 2 |
| P05093 | CYP17A1 | Steroid 17-alpha-hydroxylase/17,20 lyase |
| P05177 | CYP1A2 | Cytochrome P450 1A2 |
| P11509 | CYP2A6 | Cytochrome P450 2A6 |
| P33261 | CYP2C19 | Cytochrome P450 2C19 |
| P11712 | CYP2C9 | Cytochrome P450 2C9 |
| P10635 | CYP2D6 | Cytochrome P450 2D6 |
| P08684 | CYP3A4 | Cytochrome P450 3A4 |
| P14416 | DRD2 | D(2) dopamine receptor |
| P21917 | DRD4 | D(4) dopamine receptor |
| P00533 | EGFR | Epidermal growth factor receptor |
| P08246 | ELANE | Neutrophil elastase |
| Q13822 | ENPP2 | Ectonucleotide pyrophosphatase/phosphodiesterase family member 2 |
| P03372 | ESR1 | Estrogen receptor |
| P00734 | F2 | Prothrombin |
| O00519 | FAAH | Fatty-acid amide hydrolase 1 |
| P07148 | FABP1 | Fatty acid-binding protein, liver |
| P05413 | FABP3 | Fatty acid-binding protein, heart |
| P15090 | FABP4 | Fatty acid-binding protein, adipocyte |
| Q01469 | FABP5 | Fatty acid-binding protein 5 |
| O60427 | FADS1 | Acyl-CoA |
| P49327 | FASN | Fatty acid synthase |
| P37268 | FDFT1 | Squalene synthase |
| O14842 | FFAR1 | Free fatty acid receptor 1 |
| P17948 | FLT1 | Vascular endothelial growth factor receptor 1 |
| P02774 | GC | Vitamin D-binding protein |
| P35557 | GCK | Hexokinase-4 |
| Q12879 | GRIN2A | Glutamate receptor ionotropic, NMDA 2A |
| P49841 | GSK3B | Glycogen synthase kinase-3 beta |
| Q9BY41 | HDAC8 | Histone deacetylase 8 |
| Q16665 | HIF1A | Hypoxia-inducible factor 1-alpha |
| P04035 | HMGCR | 3-hydroxy-3-methylglutaryl-coenzyme A reductase |
| P09601 | HMOX1 | Heme oxygenase 1 |
| P28845 | HSD11B1 | Corticosteroid 11-beta-dehydrogenase isozyme 1 |
| P07900 | HSP90AA1 | Heat shock protein HSP 90-alpha |
| P28223 | HTR2A | 5-hydroxytryptamine receptor 2A |
| O75874 | IDH1 | Isocitrate dehydrogenase [NADP] cytoplasmic |
| P08069 | IGF1R | Insulin-like growth factor 1 receptor |
| O14920 | IKBKB | Inhibitor of nuclear factor kappa-B kinase subunit beta |
| P01584 | IL1B | Interleukin-1 beta |
| P40189 | IL6ST | Interleukin-6 receptor subunit beta |
| P06213 | INSR | Insulin receptor |
| P05412 | JUN | Transcription factor AP-1 |
| P21397 | MAOA | Amine oxidase [flavin-containing] A |
| P27338 | MAOB | Amine oxidase [flavin-containing] B |
| P45983 | MAPK8 | Mitogen-activated protein kinase 8 |
| P45984 | MAPK9 | Mitogen-activated protein kinase 9 |
| Q99705 | MCHR1 | Melanin-concentrating hormone receptor 1 |
| Q12866 | MERTK | Tyrosine-protein kinase Mer |
| P03956 | MMP1 | Interstitial collagenase |
| P08253 | MMP2 | 72 kDa type IV collagenase |
| P05164 | MPO | Myeloperoxidase |
| P43490 | NAMPT | Nicotinamide phosphoribosyltransferase |
| P35228 | NOS2 | Nitric oxide synthase, inducible |
| P29474 | NOS3 | Nitric oxide synthase, endothelial |
| Q9UHC9 | NPC1L1 | NPC1-like intracellular cholesterol transporter 1 |
| P55055 | NR1H2 | Oxysterols receptor LXR-beta |
| Q13133 | NR1H3 | Oxysterols receptor LXR-alpha |
| Q96RI1 | NR1H4 | Bile acid receptor |
| O75469 | NR1I2 | Nuclear receptor subfamily 1 group I member 2 |
| Q14994 | NR1I3 | Nuclear receptor subfamily 1 group I member 3 |
| P41143 | OPRD1 | Delta-type opioid receptor |
| P35372 | OPRM1 | Mu-type opioid receptor |
| Q99572 | P2RX7 | P2X purinoceptor 7 |
| P09874 | PARP1 | Poly [ADP-ribose] polymerase 1 |
| Q13370 | PDE3B | cGMP-inhibited 3',5'-cyclic phosphodiesterase B |
| O15530 | PDPK1 | 3-phosphoinositide-dependent protein kinase 1 |
| Q16875 | PFKFB3 | 6-phosphofructo-2-kinase/fructose-2,6-bisphosphatase 3 |
| P42336 | PIK3CA | Phosphatidylinositol 4,5-bisphosphate 3-kinase catalytic subunit alpha isoform |
| P14555 | PLA2G2A | Phospholipase A2, membrane associated |
| Q07869 | PPARA | Peroxisome proliferator-activated receptor alpha |
| Q03181 | PPARD | Peroxisome proliferator-activated receptor delta |
| P37231 | PPARG | Peroxisome proliferator-activated receptor gamma |
| P48147 | PREP | Prolyl endopeptidase |
| Q05655 | PRKCD | Protein kinase C delta type |
| Q02156 | PRKCE | Protein kinase C epsilon type |
| Q05513 | PRKCZ | Protein kinase C zeta type |
| P78527 | PRKDC | DNA-dependent protein kinase catalytic subunit |
| P49810 | PSEN2 | Presenilin-2 |
| P35354 | PTGS2 | Prostaglandin G/H synthase 2 |
| P18031 | PTPN1 | Tyrosine-protein phosphatase non-receptor type 1 |
| Q06124 | PTPN11 | Tyrosine-protein phosphatase non-receptor type 11 |
| P06737 | PYGL | Glycogen phosphorylase, liver form |
| P02753 | RBP4 | Retinol-binding protein 4 |
| P00797 | REN | Renin |
| Q13546 | RIPK1 | Receptor-interacting serine/threonine-protein kinase 1 |
| Q13464 | ROCK1 | Rho-associated protein kinase 1 |
| O00767 | SCD | Acyl-CoA desaturase |
| Q15393 | SF3B3 | Splicing factor 3B subunit 3 |
| P04278 | SHBG | Sex hormone-binding globulin |
| Q15465 | SHH | Sonic hedgehog protein |
| Q6P1M0 | SLC27A4 | Long-chain fatty acid transport protein 4 |
| Q01959 | SLC6A3 | Sodium-dependent dopamine transporter |
| P31645 | SLC6A4 | Sodium-dependent serotonin transporter |
| Q12772 | SREBF2 | Sterol regulatory element-binding protein 2 |
| P40763 | STAT3 | Signal transducer and activator of transcription 3 |
| O14746 | TERT | Telomerase reverse transcriptase |
| P36897 | TGFBR1 | TGF-beta receptor type-1 |
| P02766 | TTR | Transthyretin |
| P11473 | VDR | Vitamin D3 receptor |

The protein names were retrieved in Uniprot database

**Table S3.** Results of KEGG pathway analysis and GO enrichment analysis

| Item | Description | Log10(q) | Gene Rtio | Source |
| --- | --- | --- | --- | --- |
| KEGG analysis | Insulin resistance | -20.56 | 20.00 | ZX |
| KEGG analysis | Insulin signaling pathway | -13.93 | 16.47 | ZX |
| KEGG analysis | Pathways in cancer | -12.52 | 21.18 | ZX |
| KEGG analysis | Type II diabetes mellitus | -11.22 | 10.59 | ZX |
| KEGG analysis | AMPK signaling pathway | -10.40 | 12.94 | ZX |
| KEGG analysis | PPAR signaling pathway | -9.54 | 10.59 | ZX |
| KEGG analysis | Neurotrophin signaling pathway | -9.07 | 11.76 | ZX |
| KEGG analysis | Prostate cancer | -8.90 | 10.59 | ZX |
| KEGG analysis | Hepatitis C | -8.77 | 11.76 | ZX |
| KEGG analysis | FoxO signaling pathway | -8.77 | 11.76 | ZX |
| KEGG analysis | IL-17 signaling pathway | -8.77 | 10.59 | ZX |
| KEGG analysis | Endocrine resistance | -8.69 | 10.59 | ZX |
| KEGG analysis | AGE-RAGE signaling pathway in diabetic complications | -8.60 | 10.59 | ZX |
| KEGG analysis | Epithelial cell signaling in Helicobacter pylori infection | -8.46 | 9.41 | ZX |
| KEGG analysis | Prolactin signaling pathway | -8.39 | 9.41 | ZX |
| KEGG analysis | Ras signaling pathway | -7.89 | 12.94 | ZX |
| KEGG analysis | Apoptosis | -7.42 | 10.59 | ZX |
| KEGG analysis | Fluid shear stress and atherosclerosis | -7.33 | 10.59 | ZX |
| KEGG analysis | Estrogen signaling pathway | -7.32 | 9.41 | ZX |
| KEGG analysis | Focal adhesion | -7.32 | 11.76 | ZX |
| KEGG analysis | Insulin resistance | -13.25 | 14.61 | BZ |
| KEGG analysis | Pathways in cancer | -13.20 | 21.35 | BZ |
| KEGG analysis | PPAR signaling pathway | -12.47 | 12.36 | BZ |
| KEGG analysis | AGE-RAGE signaling pathway in diabetic complications | -9.47 | 11.24 | BZ |
| KEGG analysis | HIF-1 signaling pathway | -9.47 | 11.24 | BZ |
| KEGG analysis | Drug metabolism - cytochrome P450 | -9.47 | 10.11 | BZ |
| KEGG analysis | Endocrine resistance | -8.27 | 10.11 | BZ |
| KEGG analysis | Pancreatic cancer | -8.27 | 8.99 | BZ |
| KEGG analysis | Chagas disease | -8.14 | 10.11 | BZ |
| KEGG analysis | Proteoglycans in cancer | -7.98 | 12.36 | BZ |
| KEGG analysis | Serotonergic synapse | -7.82 | 10.11 | BZ |
| KEGG analysis | Chemical carcinogenesis | -7.57 | 8.99 | BZ |
| KEGG analysis | Hepatitis C | -7.32 | 10.11 | BZ |
| KEGG analysis | FoxO signaling pathway | -7.32 | 10.11 | BZ |
| KEGG analysis | Apoptosis | -7.18 | 10.11 | BZ |
| KEGG analysis | TNF signaling pathway | -6.73 | 8.99 | BZ |
| KEGG analysis | Prolactin signaling pathway | -6.71 | 7.87 | BZ |
| KEGG analysis | Metabolism of xenobiotics by cytochrome P450 | -6.56 | 7.87 | BZ |
| KEGG analysis | Osteoclast differentiation | -6.17 | 8.99 | BZ |
| KEGG analysis | Focal adhesion | -5.93 | 10.11 | BZ |
| GO.MF analysis | nuclear receptor activity | -11.78 | 11.76 | ZX |
| GO.MF analysis | transcription factor activity, direct ligand regulated sequence-specific DNA binding | -11.78 | 11.76 | ZX |
| GO.MF analysis | steroid hormone receptor activity | -11.13 | 11.76 | ZX |
| GO.MF analysis | insulin receptor substrate binding | -9.44 | 7.06 | ZX |
| GO.MF analysis | nuclear receptor transcription coactivator activity | -8.72 | 10.59 | ZX |
| GO.MF analysis | phosphotransferase activity, alcohol group as acceptor | -8.53 | 22.35 | ZX |
| GO.MF analysis | monocarboxylic acid binding | -8.36 | 10.59 | ZX |
| GO.MF analysis | transcription factor binding | -8.21 | 21.18 | ZX |
| GO.MF analysis | kinase activity | -8.05 | 22.35 | ZX |
| GO.MF analysis | protein kinase activity | -7.88 | 20.00 | ZX |
| GO.MF analysis | carboxylic acid binding | -5.67 | 11.76 | ZX |
| GO.MF analysis | lipid binding | -5.59 | 18.82 | ZX |
| GO.MF analysis | organic acid binding | -5.52 | 11.76 | ZX |
| GO.MF analysis | steroid binding | -4.86 | 8.24 | ZX |
| GO.MF analysis | protein serine/threonine kinase activity | -4.83 | 14.12 | ZX |
| GO.MF analysis | hormone binding | -4.61 | 8.24 | ZX |
| GO.MF analysis | fatty acid binding | -4.25 | 5.88 | ZX |
| GO.MF analysis | transcription coactivator activity | -4.25 | 11.76 | ZX |
| GO.MF analysis | ammonium ion binding | -4.11 | 7.06 | ZX |
| GO.MF analysis | retinoid X receptor binding | -4.04 | 4.71 | ZX |
| GO.MF analysis | nuclear receptor activity | -11.57 | 11.24 | BZ |
| GO.MF analysis | transcription factor activity, direct ligand regulated sequence-specific DNA binding | -11.57 | 11.24 | BZ |
| GO.MF analysis | oxidoreductase activity, acting on paired donors, with incorporation or reduction of molecular oxyge | -10.41 | 14.61 | BZ |
| GO.MF analysis | heme binding | -10.01 | 13.48 | BZ |
| GO.MF analysis | tetrapyrrole binding | -9.76 | 13.48 | BZ |
| GO.MF analysis | monocarboxylic acid binding | -9.76 | 11.24 | BZ |
| GO.MF analysis | lipid binding | -9.73 | 23.60 | BZ |
| GO.MF analysis | steroid hormone receptor activity | -9.54 | 10.11 | BZ |
| GO.MF analysis | oxidoreductase activity | -8.91 | 22.47 | BZ |
| GO.MF analysis | carboxylic acid binding | -7.89 | 13.48 | BZ |
| GO.MF analysis | cofactor binding | -7.72 | 17.98 | BZ |
| GO.MF analysis | organic acid binding | -7.70 | 13.48 | BZ |
| GO.MF analysis | steroid binding | -7.62 | 10.11 | BZ |
| GO.MF analysis | steroid hydroxylase activity | -7.62 | 7.87 | BZ |
| GO.MF analysis | fatty acid binding | -7.62 | 7.87 | BZ |
| GO.MF analysis | monooxygenase activity | -7.46 | 10.11 | BZ |
| GO.MF analysis | nuclear receptor transcription coactivator activity | -7.41 | 8.99 | BZ |
| GO.MF analysis | hormone receptor binding | -6.39 | 11.24 | BZ |
| GO.MF analysis | transcription factor binding | -6.33 | 17.98 | BZ |
| GO.MF analysis | oxidoreductase activity, acting on paired donors, with incorporation or reduction of molecular oxyge | -6.16 | 6.74 | BZ |
| GO.BP analysis | regulation of small molecule metabolic process | -20.85 | 31.76 | ZX |
| GO.BP analysis | cellular response to hormone stimulus | -20.13 | 35.29 | ZX |
| GO.BP analysis | regulation of lipid metabolic process | -17.06 | 27.06 | ZX |
| GO.BP analysis | cellular response to organonitrogen compound | -14.91 | 28.24 | ZX |
| GO.BP analysis | response to peptide | -14.87 | 27.06 | ZX |
| GO.BP analysis | lipid transport | -14.53 | 23.53 | ZX |
| GO.BP analysis | monocarboxylic acid metabolic process | -14.30 | 28.24 | ZX |
| GO.BP analysis | cellular response to nitrogen compound | -14.22 | 28.24 | ZX |
| GO.BP analysis | response to lipopolysaccharide | -14.01 | 22.35 | ZX |
| GO.BP analysis | positive regulation of small molecule metabolic process | -13.91 | 17.65 | ZX |
| GO.BP analysis | lipid localization | -13.91 | 23.53 | ZX |
| GO.BP analysis | regulation of lipid biosynthetic process | -13.77 | 18.82 | ZX |
| GO.BP analysis | response to molecule of bacterial origin | -13.70 | 22.35 | ZX |
| GO.BP analysis | lipid biosynthetic process | -13.67 | 28.24 | ZX |
| GO.BP analysis | response to peptide hormone | -13.29 | 23.53 | ZX |
| GO.BP analysis | response to insulin | -13.02 | 20.00 | ZX |
| GO.BP analysis | regulation of establishment of protein localization | -13.00 | 28.24 | ZX |
| GO.BP analysis | organophosphate biosynthetic process | -12.86 | 27.06 | ZX |
| GO.BP analysis | regulation of protein transport | -12.55 | 27.06 | ZX |
| GO.BP analysis | response to bacterium | -12.52 | 27.06 | ZX |
| GO.BP analysis | monocarboxylic acid metabolic process | -22.90 | 35.96 | BZ |
| GO.BP analysis | regulation of hormone levels | -21.66 | 32.58 | BZ |
| GO.BP analysis | fatty acid metabolic process | -21.62 | 29.21 | BZ |
| GO.BP analysis | steroid metabolic process | -20.38 | 26.97 | BZ |
| GO.BP analysis | lipid biosynthetic process | -19.79 | 33.71 | BZ |
| GO.BP analysis | regulation of small molecule metabolic process | -19.69 | 29.21 | BZ |
| GO.BP analysis | lipid transport | -19.69 | 26.97 | BZ |
| GO.BP analysis | regulation of lipid metabolic process | -19.69 | 28.09 | BZ |
| GO.BP analysis | lipid localization | -18.67 | 26.97 | BZ |
| GO.BP analysis | hormone metabolic process | -17.91 | 22.47 | BZ |
| GO.BP analysis | organic hydroxy compound metabolic process | -16.93 | 28.09 | BZ |
| GO.BP analysis | response to lipopolysaccharide | -16.42 | 23.60 | BZ |
| GO.BP analysis | response to molecule of bacterial origin | -15.94 | 23.60 | BZ |
| GO.BP analysis | cellular response to hormone stimulus | -15.47 | 29.21 | BZ |
| GO.BP analysis | response to nutrient levels | -15.47 | 25.84 | BZ |
| GO.BP analysis | positive regulation of small molecule metabolic process | -15.34 | 17.98 | BZ |
| GO.BP analysis | fatty acid transport | -15.34 | 15.73 | BZ |
| GO.BP analysis | response to extracellular stimulus | -14.94 | 25.84 | BZ |
| GO.BP analysis | positive regulation of lipid metabolic process | -14.24 | 16.85 | BZ |
| GO.BP analysis | response to bacterium | -14.24 | 28.09 | BZ |
| GO.CC analysis | membrane raft | -6.95 | 15.29 | ZX |
| GO.CC analysis | membrane microdomain | -6.95 | 15.29 | ZX |
| GO.CC analysis | membrane region | -6.92 | 15.29 | ZX |
| GO.CC analysis | RNA polymerase II transcription factor complex | -6.90 | 11.76 | ZX |
| GO.CC analysis | nuclear transcription factor complex | -6.08 | 11.76 | ZX |
| GO.CC analysis | integral component of presynaptic membrane | -5.63 | 8.24 | ZX |
| GO.CC analysis | intrinsic component of presynaptic membrane | -5.34 | 8.24 | ZX |
| GO.CC analysis | neuronal cell body | -5.24 | 15.29 | ZX |
| GO.CC analysis | receptor complex | -5.18 | 15.29 | ZX |
| GO.CC analysis | axon | -5.09 | 16.47 | ZX |
| GO.CC analysis | transcription factor complex | -5.05 | 12.94 | ZX |
| GO.CC analysis | integral component of synaptic membrane | -4.96 | 9.41 | ZX |
| GO.CC analysis | presynaptic membrane | -4.95 | 9.41 | ZX |
| GO.CC analysis | cell body | -4.87 | 15.29 | ZX |
| GO.CC analysis | intrinsic component of synaptic membrane | -4.81 | 9.41 | ZX |
| GO.CC analysis | postsynapse | -4.44 | 15.29 | ZX |
| GO.CC analysis | dendrite | -4.44 | 15.29 | ZX |
| GO.CC analysis | dendritic tree | -4.44 | 15.29 | ZX |
| GO.CC analysis | vesicle lumen | -3.54 | 10.59 | ZX |
| GO.CC analysis | integral component of postsynaptic membrane | -3.48 | 7.06 | ZX |
| GO.CC analysis | membrane raft | -9.02 | 16.85 | BZ |
| GO.CC analysis | membrane microdomain | -9.02 | 16.85 | BZ |
| GO.CC analysis | membrane region | -8.96 | 16.85 | BZ |
| GO.CC analysis | RNA polymerase II transcription factor complex | -8.05 | 12.36 | BZ |
| GO.CC analysis | nuclear transcription factor complex | -7.13 | 12.36 | BZ |
| GO.CC analysis | transcription factor complex | -6.67 | 14.61 | BZ |
| GO.CC analysis | integral component of presynaptic membrane | -5.56 | 7.87 | BZ |
| GO.CC analysis | intrinsic component of presynaptic membrane | -5.26 | 7.87 | BZ |
| GO.CC analysis | caveola | -3.89 | 6.74 | BZ |
| GO.CC analysis | integral component of synaptic membrane | -3.52 | 7.87 | BZ |
| GO.CC analysis | presynaptic membrane | -3.52 | 7.87 | BZ |
| GO.CC analysis | intrinsic component of synaptic membrane | -3.38 | 7.87 | BZ |
| GO.CC analysis | plasma membrane raft | -3.28 | 6.74 | BZ |
| GO.CC analysis | receptor complex | -2.55 | 11.24 | BZ |
| GO.CC analysis | vacuolar lumen | -2.27 | 6.74 | BZ |
| GO.CC analysis | integral component of postsynaptic membrane | -2.07 | 5.62 | BZ |
| GO.CC analysis | intrinsic component of postsynaptic membrane | -2.01 | 5.62 | BZ |
| GO.CC analysis | axon | -1.95 | 11.24 | BZ |
| GO.CC analysis | postsynapse | -1.95 | 11.24 | BZ |
| GO.CC analysis | presynapse | -1.92 | 10.11 | BZ |

The values were rounded up to 2 decimal places

**Table S4.** Hit targets of lipid metabolic biological process in ZX and BZ

| Uniprot ID | Gene symbol | Protein name | Source |
| --- | --- | --- | --- |
| O00763 | ACACB | Acetyl-CoA carboxylase 2 | ZX |
| P00734 | F2 | Prothrombin | ZX |
| P49327 | FASN | Fatty acid synthase | ZX |
| P37268 | FDFT1 | Squalene synthase | ZX |
| P04035 | HMGCR | 3-hydroxy-3-methylglutaryl-coenzyme A reductase | ZX |
| P28223 | HTR2A | 5-hydroxytryptamine receptor 2A | ZX |
| O75874 | IDH1 | Isocitrate dehydrogenase [NADP] cytoplasmic | ZX |
| Q96RI1 | NR1H4 | Bile acid receptor | ZX |
| Q13370 | PDE3B | cGMP-inhibited 3',5'-cyclic phosphodiesterase B | ZX |
| Q05655 | PRKCD | Protein kinase C delta type | ZX |
| Q02156 | PRKCE | Protein kinase C epsilon type | ZX |
| Q12772 | SREBF2 | Sterol regulatory element-binding protein 2 | ZX |
| P11473 | VDR | Vitamin D3 receptor | ZX |
| P21554 | CNR1 | Cannabinoid receptor 1 | ZX and BZ |
| P05413 | FABP3 | Fatty acid-binding protein, heart | ZX and BZ |
| Q01469 | FABP5 | Fatty acid-binding protein 5 | ZX and BZ |
| P55055 | NR1H2 | Oxysterols receptor LXR-beta | ZX and BZ |
| Q13133 | NR1H3 | Oxysterols receptor LXR-alpha | ZX and BZ |
| Q07869 | PPARA | Peroxisome proliferator-activated receptor alpha | ZX and BZ |
| Q03181 | PPARD | Peroxisome proliferator-activated receptor delta | ZX and BZ |
| P37231 | PPARG | Peroxisome proliferator-activated receptor gamma | ZX and BZ |
| P35354 | PTGS2 | Prostaglandin G/H synthase 2 | ZX and BZ |
| O00767 | SCD | Acyl-CoA desaturase | ZX and BZ |
| P31749 | AKT1 | RAC-alpha serine/threonine-protein kinase | BZ |
| P18054 | ALOX12 | Arachidonate 12-lipoxygenase, 12S-type | BZ |
| P19835 | CEL | Bile salt-activated lipase | BZ |
| P23141 | CES1 | Liver carboxylesterase 1 | BZ |
| O00748 | CES2 | Cocaine esterase | BZ |
| P05177 | CYP1A2 | Cytochrome P450 1A2 | BZ |
| P11509 | CYP2A6 | Cytochrome P450 2A6 | BZ |
| P33261 | CYP2C19 | Cytochrome P450 2C19 | BZ |
| P11712 | CYP2C9 | Cytochrome P450 2C9 | BZ |
| P10635 | CYP2D6 | Cytochrome P450 2D6 | BZ |
| P08684 | CYP3A4 | Cytochrome P450 3A4 | BZ |
| O00519 | FAAH | Fatty-acid amide hydrolase 1 | BZ |
| P07148 | FABP1 | Fatty acid-binding protein, liver | BZ |
| O60427 | FADS1 | Acyl-CoA | BZ |
| P01584 | IL1B | Interleukin-1 beta | BZ |
| Q6P1M0 | SLC27A4 | Long-chain fatty acid transport protein 4 | BZ |

| **Table S5.** Results of PPI analysis by STRING platform | | |
| --- | --- | --- |
| Target 1 | Target 2 | Combine score |
| RAC-alpha serine/threonine-protein kinase | Glycogen synthase kinase-3 beta | 0.999 |
| RAC-alpha serine/threonine-protein kinase | Nitric oxide synthase, endothelial | 0.999 |
| Mitogen-activated protein kinase 9 | Transcription factor AP-1 | 0.999 |
| RAC-alpha serine/threonine-protein kinase | 3-phosphoinositide-dependent protein kinase 1 | 0.999 |
| Tyrosine-protein phosphatase non-receptor type 1 | Insulin-like growth factor 1 receptor | 0.999 |
| Mitogen-activated protein kinase 8 | Transcription factor AP-1 | 0.999 |
| RAC-alpha serine/threonine-protein kinase | Phosphatidylinositol 4,5-bisphosphate 3-kinase catalytic subunit alpha isoform | 0.998 |
| Epidermal growth factor receptor | Signal transducer and activator of transcription 3 | 0.998 |
| RAC-alpha serine/threonine-protein kinase | Heat shock protein HSP 90-alpha | 0.998 |
| Tyrosine-protein phosphatase non-receptor type 1 | Insulin receptor | 0.998 |
| Retinol-binding protein 4 | Transthyretin | 0.998 |
| Tyrosine-protein phosphatase non-receptor type 11 | Epidermal growth factor receptor | 0.996 |
| Neutrophil elastase | Myeloperoxidase | 0.995 |
| Insulin receptor | Insulin-like growth factor 1 receptor | 0.994 |
| RAC-alpha serine/threonine-protein kinase | Signal transducer and activator of transcription 3 | 0.994 |
| Signal transducer and activator of transcription 3 | G1/S-specific cyclin-D1 | 0.993 |
| Tyrosine-protein phosphatase non-receptor type 11 | Signal transducer and activator of transcription 3 | 0.991 |
| RAC-alpha serine/threonine-protein kinase | Estrogen receptor | 0.991 |
| Inhibitor of nuclear factor kappa-B kinase subunit beta | Inhibitor of nuclear factor kappa-B kinase subunit alpha | 0.991 |
| Peroxisome proliferator-activated receptor gamma | Fatty acid-binding protein, adipocyte | 0.991 |
| Heat shock protein HSP 90-alpha | Nitric oxide synthase, endothelial | 0.991 |
| Estrogen receptor | G1/S-specific cyclin-D1 | 0.990 |
| Glycogen synthase kinase-3 beta | G1/S-specific cyclin-D1 | 0.990 |
| Epidermal growth factor receptor | Phosphatidylinositol 4,5-bisphosphate 3-kinase catalytic subunit alpha isoform | 0.990 |
| D(2) dopamine receptor | Sodium-dependent dopamine transporter | 0.990 |
| Heat shock protein HSP 90-alpha | Epidermal growth factor receptor | 0.989 |
| Estrogen receptor | Insulin-like growth factor 1 receptor | 0.989 |
| RAC-alpha serine/threonine-protein kinase | DNA-dependent protein kinase catalytic subunit | 0.988 |
| Acetyl-CoA carboxylase 2 | Fatty acid synthase | 0.988 |
| Peroxisome proliferator-activated receptor alpha | Fatty acid-binding protein, liver | 0.988 |
| RAC-alpha serine/threonine-protein kinase | Hypoxia-inducible factor 1-alpha | 0.988 |
| Tyrosine-protein phosphatase non-receptor type 1 | Epidermal growth factor receptor | 0.988 |
| Interleukin-6 receptor subunit beta | Signal transducer and activator of transcription 3 | 0.987 |
| RAC-alpha serine/threonine-protein kinase | Tyrosine-protein phosphatase non-receptor type 1 | 0.987 |
| Hypoxia-inducible factor 1-alpha | Signal transducer and activator of transcription 3 | 0.986 |
| Estrogen receptor | Nitric oxide synthase, endothelial | 0.985 |
| RAC-alpha serine/threonine-protein kinase | Telomerase reverse transcriptase | 0.985 |
| Transcription factor AP-1 | G1/S-specific cyclin-D1 | 0.985 |
| Mitogen-activated protein kinase 8 | Amyloid-beta precursor protein | 0.984 |
| D(2) dopamine receptor | D(2) dopamine receptor | 0.984 |
| Insulin-like growth factor 1 receptor | Phosphatidylinositol 4,5-bisphosphate 3-kinase catalytic subunit alpha isoform | 0.984 |
| Cannabinoid receptor 1 | Delta-type opioid receptor | 0.983 |
| Heat shock protein HSP 90-alpha | Telomerase reverse transcriptase | 0.982 |
| Heat shock protein HSP 90-alpha | Signal transducer and activator of transcription 3 | 0.982 |
| Presenilin-2 | Amyloid-beta precursor protein | 0.981 |
| Prostaglandin G/H synthase 2 | Signal transducer and activator of transcription 3 | 0.980 |
| Estrogen receptor | Transcription factor AP-1 | 0.979 |
| Estrogen receptor | Heat shock protein HSP 90-alpha | 0.979 |
| Signal transducer and activator of transcription 3 | 72 kDa type IV collagenase | 0.979 |
| Transcription factor AP-1 | Glycogen synthase kinase-3 beta | 0.978 |
| Cytochrome P450 2C19 | Cytochrome P450 2C9 | 0.977 |
| Transcription factor AP-1 | Heme oxygenase 1 | 0.977 |
| Estrogen receptor | Phosphatidylinositol 4,5-bisphosphate 3-kinase catalytic subunit alpha isoform | 0.977 |
| Hypoxia-inducible factor 1-alpha | Heat shock protein HSP 90-alpha | 0.976 |
| Tyrosine-protein phosphatase non-receptor type 11 | Vascular endothelial growth factor receptor 1 | 0.976 |
| RAC-alpha serine/threonine-protein kinase | Tyrosine-protein phosphatase non-receptor type 11 | 0.976 |
| RAC-alpha serine/threonine-protein kinase | Inhibitor of nuclear factor kappa-B kinase subunit alpha | 0.975 |
| RAC-alpha serine/threonine-protein kinase | Inhibitor of nuclear factor kappa-B kinase subunit beta | 0.974 |
| Prostaglandin G/H synthase 2 | Interleukin-1 beta | 0.974 |
| Hypoxia-inducible factor 1-alpha | Epidermal growth factor receptor | 0.973 |
| 3-phosphoinositide-dependent protein kinase 1 | Phosphatidylinositol 4,5-bisphosphate 3-kinase catalytic subunit alpha isoform | 0.973 |
| Cytochrome P450 3A4 | Cytochrome P450 2C9 | 0.970 |
| Amyloid-beta precursor protein | Transthyretin | 0.970 |
| Hypoxia-inducible factor 1-alpha | Transcription factor AP-1 | 0.970 |
| Hypoxia-inducible factor 1-alpha | Nitric oxide synthase, endothelial | 0.969 |
| Fatty acid-binding protein, liver | Fatty acid-binding protein, adipocyte | 0.969 |
| Estrogen receptor | Telomerase reverse transcriptase | 0.968 |
| Squalene synthase | Sterol regulatory element-binding protein 2 | 0.967 |
| Mitogen-activated protein kinase 9 | Mitogen-activated protein kinase 8 | 0.967 |
| Tyrosine-protein phosphatase non-receptor type 11 | Insulin-like growth factor 1 receptor | 0.967 |
| Peroxisome proliferator-activated receptor alpha | Acyl-CoA | 0.966 |
| Nuclear receptor subfamily 1 group I member 3 | Cytochrome P450 1A2 | 0.966 |
| Interstitial collagenase | Signal transducer and activator of transcription 3 | 0.966 |
| Signal transducer and activator of transcription 3 | Interleukin-1 beta | 0.965 |
| Cannabinoid receptor 2 | Delta-type opioid receptor | 0.965 |
| D(4) dopamine receptor | D(4) dopamine receptor | 0.965 |
| Interleukin-6 receptor subunit beta | Tyrosine-protein phosphatase non-receptor type 11 | 0.965 |
| Type-1 angiotensin II receptor | Epidermal growth factor receptor | 0.964 |
| Transcription factor AP-1 | Interleukin-1 beta | 0.963 |
| Cytochrome P450 3A4 | Nuclear receptor subfamily 1 group I member 2 | 0.962 |
| Protein kinase C zeta type | Glycogen synthase kinase-3 beta | 0.960 |
| Angiotensin-converting enzyme | Renin | 0.959 |
| Mitogen-activated protein kinase 9 | Amyloid-beta precursor protein | 0.959 |
| Liver carboxylesterase 1 | Cytochrome P450 3A4 | 0.958 |
| RAC-alpha serine/threonine-protein kinase | cGMP-inhibited 3',5'-cyclic phosphodiesterase B | 0.958 |
| Protein kinase C delta type | 3-phosphoinositide-dependent protein kinase 1 | 0.957 |
| 3-phosphoinositide-dependent protein kinase 1 | Heat shock protein HSP 90-alpha | 0.956 |
| Transcription factor AP-1 | Peroxisome proliferator-activated receptor gamma | 0.956 |
| Tyrosine-protein phosphatase non-receptor type 11 | Insulin receptor | 0.956 |
| Protein kinase C delta type | Signal transducer and activator of transcription 3 | 0.956 |
| Transcription factor AP-1 | Nitric oxide synthase, inducible | 0.956 |
| Peroxisome proliferator-activated receptor alpha | Heat shock protein HSP 90-alpha | 0.956 |
| Cannabinoid receptor 1 | Fatty-acid amide hydrolase 1 | 0.956 |
| Cytochrome P450 3A4 | Cocaine esterase | 0.956 |
| 3-phosphoinositide-dependent protein kinase 1 | Protein kinase C epsilon type | 0.956 |
| Type-1 angiotensin II receptor | Renin | 0.955 |
| Nitric oxide synthase, inducible | Signal transducer and activator of transcription 3 | 0.955 |
| Protein kinase C delta type | Phosphatidylinositol 4,5-bisphosphate 3-kinase catalytic subunit alpha isoform | 0.954 |
| Vascular endothelial growth factor receptor 1 | Phosphatidylinositol 4,5-bisphosphate 3-kinase catalytic subunit alpha isoform | 0.954 |
| Protein kinase C zeta type | 3-phosphoinositide-dependent protein kinase 1 | 0.953 |
| Sterol regulatory element-binding protein 2 | Peroxisome proliferator-activated receptor gamma | 0.953 |
| Sterol regulatory element-binding protein 2 | 3-hydroxy-3-methylglutaryl-coenzyme A reductase | 0.953 |
| Transcription factor AP-1 | Nitric oxide synthase, endothelial | 0.952 |
| Protein kinase C delta type | Nitric oxide synthase, endothelial | 0.952 |
| Cytochrome P450 1A2 | Cytochrome P450 3A4 | 0.952 |
| Cytochrome P450 2C19 | Cytochrome P450 3A4 | 0.951 |
| D(2) dopamine receptor | D(2) dopamine receptor | 0.951 |
| Cytochrome P450 2D6 | Amine oxidase [flavin-containing] A | 0.951 |
| Prostaglandin G/H synthase 2 | Arachidonate 12-lipoxygenase, 12S-type | 0.951 |
| ALK tyrosine kinase receptor | Phosphatidylinositol 4,5-bisphosphate 3-kinase catalytic subunit alpha isoform | 0.950 |
| Inhibitor of nuclear factor kappa-B kinase subunit beta | Receptor-interacting serine/threonine-protein kinase 1 | 0.950 |
| Insulin receptor | Phosphatidylinositol 4,5-bisphosphate 3-kinase catalytic subunit alpha isoform | 0.950 |
| Oxysterols receptor LXR-alpha | Peroxisome proliferator-activated receptor alpha | 0.949 |
| Cytochrome P450 3A4 | Cytochrome P450 2A6 | 0.949 |
| D(4) dopamine receptor | D(4) dopamine receptor | 0.949 |
| Tyrosine-protein phosphatase non-receptor type 11 | Phosphatidylinositol 4,5-bisphosphate 3-kinase catalytic subunit alpha isoform | 0.949 |
| Peroxisome proliferator-activated receptor delta | Fatty acid-binding protein 5 | 0.948 |
| RAC-alpha serine/threonine-protein kinase | Insulin receptor | 0.948 |
| Cytochrome P450 2D6 | Cytochrome P450 3A4 | 0.948 |
| Protein kinase C delta type | Insulin-like growth factor 1 receptor | 0.946 |
| Amine oxidase [flavin-containing] B | Cytochrome P450 2D6 | 0.946 |
| Inhibitor of nuclear factor kappa-B kinase subunit beta | Protein kinase C epsilon type | 0.946 |
| Mu-type opioid receptor | Delta-type opioid receptor | 0.945 |
| Amine oxidase [flavin-containing] B | Amine oxidase [flavin-containing] A | 0.945 |
| Corticosteroid 11-beta-dehydrogenase isozyme 1 | Peroxisome proliferator-activated receptor gamma | 0.945 |
| Prothrombin | Amyloid-beta precursor protein | 0.944 |
| Protein kinase C delta type | Protein kinase C zeta type | 0.944 |
| Estrogen receptor | Epidermal growth factor receptor | 0.943 |
| Signal transducer and activator of transcription 3 | Phosphatidylinositol 4,5-bisphosphate 3-kinase catalytic subunit alpha isoform | 0.943 |
| RAC-alpha serine/threonine-protein kinase | G1/S-specific cyclin-D1 | 0.943 |
| Neutrophil elastase | Interstitial collagenase | 0.943 |
| D(4) dopamine receptor | D(4) dopamine receptor | 0.943 |
| Inhibitor of nuclear factor kappa-B kinase subunit alpha | Receptor-interacting serine/threonine-protein kinase 1 | 0.943 |
| RAC-alpha serine/threonine-protein kinase | Protein kinase C zeta type | 0.942 |
| Peroxisome proliferator-activated receptor alpha | Transcription factor AP-1 | 0.938 |
| Retinol-binding protein 4 | Liver carboxylesterase 1 | 0.938 |
| Mu-type opioid receptor | Cannabinoid receptor 1 | 0.938 |
| Cannabinoid receptor 1 | Melanin-concentrating hormone receptor 1 | 0.938 |
| 3-phosphoinositide-dependent protein kinase 1 | Insulin receptor | 0.937 |
| 3-phosphoinositide-dependent protein kinase 1 | Glycogen synthase kinase-3 beta | 0.937 |
| Inhibitor of nuclear factor kappa-B kinase subunit beta | Protein kinase C zeta type | 0.936 |
| Heat shock protein HSP 90-alpha | Phosphatidylinositol 4,5-bisphosphate 3-kinase catalytic subunit alpha isoform | 0.936 |
| Squalene synthase | 3-hydroxy-3-methylglutaryl-coenzyme A reductase | 0.936 |
| Prostaglandin G/H synthase 2 | Cytochrome P450 2C9 | 0.936 |
| Steroid 17-alpha-hydroxylase/17,20 lyase | Cytochrome P450 3A4 | 0.935 |
| ATP-dependent translocase ABCB1 | Mitogen-activated protein kinase 8 | 0.935 |
| D(2) dopamine receptor | D(2) dopamine receptor | 0.932 |
| Cytochrome P450 1A2 | Cytochrome P450 2A6 | 0.932 |
| Oxysterols receptor LXR-alpha | Fatty acid-binding protein, liver | 0.932 |
| Amine oxidase [flavin-containing] A | Cytochrome P450 3A4 | 0.932 |
| Cytochrome P450 1A2 | Cytochrome P450 2C9 | 0.931 |
| Type-1 angiotensin II receptor | Prothrombin | 0.931 |
| D(2) dopamine receptor | Amyloid-beta precursor protein | 0.931 |
| Cytochrome P450 2C19 | Cytochrome P450 1A2 | 0.930 |
| Rho-associated protein kinase 1 | Heat shock protein HSP 90-alpha | 0.930 |
| D(2) dopamine receptor | Delta-type opioid receptor | 0.930 |
| Corticosteroid 11-beta-dehydrogenase isozyme 1 | Cytochrome P450 3A4 | 0.929 |
| D(4) dopamine receptor | D(4) dopamine receptor | 0.929 |
| Mu-type opioid receptor | Cannabinoid receptor 2 | 0.929 |
| Type-1 angiotensin II receptor | Angiotensin-converting enzyme | 0.928 |
| Corticosteroid 11-beta-dehydrogenase isozyme 1 | Cytochrome P450 2D6 | 0.928 |
| Liver carboxylesterase 1 | Myeloperoxidase | 0.928 |
| Amine oxidase [flavin-containing] B | Cytochrome P450 2C19 | 0.928 |
| 3-phosphoinositide-dependent protein kinase 1 | Nitric oxide synthase, endothelial | 0.928 |
| D(4) dopamine receptor | D(4) dopamine receptor | 0.927 |
| Cannabinoid receptor 1 | Amyloid-beta precursor protein | 0.927 |
| Amyloid-beta precursor protein | Delta-type opioid receptor | 0.926 |
| Heat shock protein HSP 90-alpha | Receptor-interacting serine/threonine-protein kinase 1 | 0.926 |
| Oxysterols receptor LXR-alpha | Transcription factor AP-1 | 0.926 |
| Protein kinase C zeta type | Tyrosine-protein phosphatase non-receptor type 11 | 0.926 |
| Cytochrome P450 2C19 | Amine oxidase [flavin-containing] A | 0.926 |
| Cytochrome P450 2C19 | Prostaglandin G/H synthase 2 | 0.926 |
| Amine oxidase [flavin-containing] B | Cytochrome P450 3A4 | 0.925 |
| Protein kinase C zeta type | Phosphatidylinositol 4,5-bisphosphate 3-kinase catalytic subunit alpha isoform | 0.924 |
| Cannabinoid receptor 2 | Melanin-concentrating hormone receptor 1 | 0.924 |
| Acyl-CoA | Fatty acid-binding protein, liver | 0.924 |
| TGF-beta receptor type-1 | 3-phosphoinositide-dependent protein kinase 1 | 0.923 |
| Tyrosine-protein phosphatase non-receptor type 1 | Phosphatidylinositol 4,5-bisphosphate 3-kinase catalytic subunit alpha isoform | 0.923 |
| Inhibitor of nuclear factor kappa-B kinase subunit alpha | 3-phosphoinositide-dependent protein kinase 1 | 0.923 |
| Tyrosine-protein phosphatase non-receptor type 11 | Nitric oxide synthase, endothelial | 0.923 |
| Squalene synthase | Peroxisome proliferator-activated receptor alpha | 0.923 |
| Mitogen-activated protein kinase 8 | Protein kinase C zeta type | 0.923 |
| Interstitial collagenase | 72 kDa type IV collagenase | 0.922 |
| 5-hydroxytryptamine receptor 2A | Amyloid-beta precursor protein | 0.922 |
| Cytochrome P450 2D6 | Cytochrome P450 2A6 | 0.922 |
| Cannabinoid receptor 2 | Fatty-acid amide hydrolase 1 | 0.922 |
| Peroxisome proliferator-activated receptor alpha | Nitric oxide synthase, inducible | 0.922 |
| Inhibitor of nuclear factor kappa-B kinase subunit beta | 3-phosphoinositide-dependent protein kinase 1 | 0.922 |
| D(4) dopamine receptor | D(4) dopamine receptor | 0.921 |
| Transthyretin | Myeloperoxidase | 0.921 |
| Transcription factor AP-1 | Signal transducer and activator of transcription 3 | 0.921 |
| Heat shock protein HSP 90-alpha | 72 kDa type IV collagenase | 0.921 |
| Steroid 17-alpha-hydroxylase/17,20 lyase | Cytochrome P450 1A2 | 0.920 |
| Isocitrate dehydrogenase [NADP] cytoplasmic | Nitric oxide synthase, inducible | 0.920 |
| Neutrophil elastase | Transthyretin | 0.919 |
| Corticosteroid 11-beta-dehydrogenase isozyme 1 | Cytochrome P450 2A6 | 0.919 |
| Type-1 angiotensin II receptor | Amyloid-beta precursor protein | 0.918 |
| Mu-type opioid receptor | Signal transducer and activator of transcription 3 | 0.918 |
| Protein kinase C zeta type | Insulin receptor | 0.918 |
| C-X-C chemokine receptor type 2 | Amyloid-beta precursor protein | 0.917 |
| Fatty acid-binding protein 5 | Transthyretin | 0.917 |
| Melanin-concentrating hormone receptor 1 | Free fatty acid receptor 1 | 0.917 |
| D(2) dopamine receptor | Melanin-concentrating hormone receptor 1 | 0.917 |
| Epidermal growth factor receptor | G1/S-specific cyclin-D1 | 0.917 |
| Cannabinoid receptor 2 | Amyloid-beta precursor protein | 0.916 |
| Mitogen-activated protein kinase 9 | Protein kinase C zeta type | 0.916 |
| Mu-type opioid receptor | Amyloid-beta precursor protein | 0.916 |
| Cytochrome P450 2C9 | Arachidonate 12-lipoxygenase, 12S-type | 0.915 |
| Phosphatidylinositol 4,5-bisphosphate 3-kinase catalytic subunit alpha isoform | Interleukin-1 beta | 0.915 |
| Cytochrome P450 2C19 | Cytochrome P450 2D6 | 0.914 |
| Cytochrome P450 2A6 | Cytochrome P450 2C9 | 0.913 |
| Inhibitor of nuclear factor kappa-B kinase subunit alpha | Protein kinase C epsilon type | 0.913 |
| RAC-alpha serine/threonine-protein kinase | Transcription factor AP-1 | 0.913 |
| Signal transducer and activator of transcription 3 | Delta-type opioid receptor | 0.912 |
| Acyl-CoA desaturase | Fatty acid synthase | 0.912 |
| Cytochrome P450 2C19 | Arachidonate 12-lipoxygenase, 12S-type | 0.911 |
| D(4) dopamine receptor | D(4) dopamine receptor | 0.911 |
| Cannabinoid receptor 2 | C-X-C chemokine receptor type 2 | 0.910 |
| Squalene synthase | Fatty acid-binding protein, liver | 0.910 |
| 5-hydroxytryptamine receptor 2A | Free fatty acid receptor 1 | 0.909 |
| 5-hydroxytryptamine receptor 2A | Melanin-concentrating hormone receptor 1 | 0.909 |
| RAC-alpha serine/threonine-protein kinase | Nitric oxide synthase, inducible | 0.909 |
| Amyloid-beta precursor protein | Phosphatidylinositol 4,5-bisphosphate 3-kinase catalytic subunit alpha isoform | 0.908 |
| Transcription factor AP-1 | Fatty acid-binding protein, liver | 0.908 |
| Mu-type opioid receptor | C-X-C chemokine receptor type 2 | 0.908 |
| Melanin-concentrating hormone receptor 1 | Delta-type opioid receptor | 0.908 |
| 5-hydroxytryptamine receptor 2A | Prothrombin | 0.908 |
| Cannabinoid receptor 2 | Cannabinoid receptor 1 | 0.908 |
| Estrogen receptor | Mitogen-activated protein kinase 8 | 0.908 |
| Peroxisome proliferator-activated receptor alpha | Prothrombin | 0.908 |
| 5-hydroxytryptamine receptor 2A | Sodium-dependent serotonin transporter | 0.907 |
| Cannabinoid receptor 1 | C-X-C chemokine receptor type 2 | 0.907 |
| Protein kinase C zeta type | Inhibitor of nuclear factor kappa-B kinase subunit alpha | 0.907 |
| Rho-associated protein kinase 1 | Protein kinase C zeta type | 0.907 |
| Mitogen-activated protein kinase 8 | Signal transducer and activator of transcription 3 | 0.906 |
| Protein kinase C delta type | Transthyretin | 0.906 |
| 5-hydroxytryptamine receptor 2A | Type-1 angiotensin II receptor | 0.906 |
| Neutrophil elastase | Fatty acid-binding protein 5 | 0.906 |
| Vitamin D3 receptor | Vitamin D-binding protein | 0.906 |
| Mu-type opioid receptor | Melanin-concentrating hormone receptor 1 | 0.906 |
| Fatty acid-binding protein 5 | Fatty acid-binding protein, adipocyte | 0.906 |
| Prostaglandin G/H synthase 2 | Nitric oxide synthase, inducible | 0.906 |
| Protein kinase C delta type | Protein kinase C epsilon type | 0.906 |
| Type-1 angiotensin II receptor | Mu-type opioid receptor | 0.905 |
| C-X-C chemokine receptor type 2 | Delta-type opioid receptor | 0.905 |
| Type-1 angiotensin II receptor | Delta-type opioid receptor | 0.905 |
| Protein kinase C delta type | Myeloperoxidase | 0.905 |
| Retinol-binding protein 4 | Cocaine esterase | 0.904 |
| Fatty acid-binding protein, heart | Fatty acid-binding protein, adipocyte | 0.904 |
| Oxysterols receptor LXR-alpha | Oxysterols receptor LXR-beta | 0.903 |
| Type-1 angiotensin II receptor | Melanin-concentrating hormone receptor 1 | 0.903 |
| Protein kinase C zeta type | TGF-beta receptor type-1 | 0.903 |
| Prothrombin | Phosphatidylinositol 4,5-bisphosphate 3-kinase catalytic subunit alpha isoform | 0.903 |
| Neutrophil elastase | Protein kinase C delta type | 0.903 |
| Sterol regulatory element-binding protein 2 | NPC1-like intracellular cholesterol transporter 1 | 0.902 |
| Fatty acid-binding protein 5 | Myeloperoxidase | 0.902 |
| Isocitrate dehydrogenase [NADP] cytoplasmic | Cathepsin S | 0.902 |
| Amyloid-beta precursor protein | Melanin-concentrating hormone receptor 1 | 0.902 |
| Type-1 angiotensin II receptor | Phosphatidylinositol 4,5-bisphosphate 3-kinase catalytic subunit alpha isoform | 0.902 |
| D(2) dopamine receptor | C-X-C chemokine receptor type 2 | 0.901 |
| Protein kinase C delta type | Sonic hedgehog protein | 0.901 |
| Type-1 angiotensin II receptor | Free fatty acid receptor 1 | 0.901 |
| Prothrombin | Free fatty acid receptor 1 | 0.900 |
| D(4) dopamine receptor | D(4) dopamine receptor | 0.900 |
| Protein kinase C zeta type | Receptor-interacting serine/threonine-protein kinase 1 | 0.900 |
| Phosphatidylinositol 4,5-bisphosphate 3-kinase catalytic subunit alpha isoform | Melanin-concentrating hormone receptor 1 | 0.900 |
| Protein kinase C delta type | Fatty acid-binding protein 5 | 0.900 |
| Prothrombin | Melanin-concentrating hormone receptor 1 | 0.900 |
| C-X-C chemokine receptor type 2 | Melanin-concentrating hormone receptor 1 | 0.900 |
| Phosphatidylinositol 4,5-bisphosphate 3-kinase catalytic subunit alpha isoform | Free fatty acid receptor 1 | 0.900 |
| Amyloid-beta precursor protein | Free fatty acid receptor 1 | 0.900 |
| 5-hydroxytryptamine receptor 2A | Phosphatidylinositol 4,5-bisphosphate 3-kinase catalytic subunit alpha isoform | 0.900 |
| The values were rounded up to 3 decimal places | | |

**Table S6.** Results of network analysis by Cytoscape network analyzer

| Gene Symbol | BetweennessCentrality | ClosenessCentrality | Degree | Radiality | Research object |
| --- | --- | --- | --- | --- | --- |
| AKT1 | 0.13 | 0.68 | 64.00 | 0.90 | All matched targets |
| PTGS2 | 0.05 | 0.60 | 45.00 | 0.87 | All matched targets |
| EGFR | 0.04 | 0.60 | 44.00 | 0.87 | All matched targets |
| STAT3 | 0.04 | 0.59 | 44.00 | 0.86 | All matched targets |
| MAPK8 | 0.03 | 0.60 | 43.00 | 0.87 | All matched targets |
| PPARG | 0.06 | 0.58 | 41.00 | 0.85 | All matched targets |
| JUN | 0.02 | 0.58 | 40.00 | 0.86 | All matched targets |
| IL1B | 0.04 | 0.58 | 39.00 | 0.86 | All matched targets |
| HSP90AA1 | 0.03 | 0.57 | 37.00 | 0.85 | All matched targets |
| ESR1 | 0.05 | 0.56 | 36.00 | 0.84 | All matched targets |
| APP | 0.05 | 0.58 | 36.00 | 0.86 | All matched targets |
| NOS3 | 0.02 | 0.56 | 33.00 | 0.85 | All matched targets |
| CCND1 | 0.01 | 0.54 | 30.00 | 0.83 | All matched targets |
| CYP3A4 | 0.03 | 0.53 | 27.00 | 0.82 | All matched targets |
| PPARA | 0.03 | 0.55 | 27.00 | 0.84 | All matched targets |
| PIK3CA | 0.02 | 0.53 | 27.00 | 0.83 | All matched targets |
| HIF1A | 0.01 | 0.53 | 27.00 | 0.83 | All matched targets |
| F2 | 0.02 | 0.54 | 26.00 | 0.83 | All matched targets |
| ACE | 0.01 | 0.54 | 26.00 | 0.83 | All matched targets |
| IGF1R | 0.01 | 0.55 | 26.00 | 0.84 | All matched targets |
| MMP2 | 0.00 | 0.53 | 25.00 | 0.83 | All matched targets |
| PRKCD | 0.02 | 0.49 | 24.00 | 0.79 | All matched targets |
| REN | 0.02 | 0.53 | 24.00 | 0.82 | All matched targets |
| FASN | 0.02 | 0.50 | 24.00 | 0.80 | All matched targets |
| OPRM1 | 0.01 | 0.52 | 24.00 | 0.81 | All matched targets |
| ABCB1 | 0.02 | 0.52 | 22.00 | 0.82 | All matched targets |
| HMGCR | 0.02 | 0.52 | 21.00 | 0.82 | All matched targets |
| MPO | 0.01 | 0.52 | 21.00 | 0.82 | All matched targets |
| HMOX1 | 0.00 | 0.52 | 21.00 | 0.81 | All matched targets |
| FABP1 | 0.01 | 0.48 | 20.00 | 0.78 | All matched targets |
| SCD | 0.01 | 0.49 | 20.00 | 0.79 | All matched targets |
| AGTR1 | 0.01 | 0.52 | 20.00 | 0.81 | All matched targets |
| CNR1 | 0.01 | 0.51 | 20.00 | 0.81 | All matched targets |
| PTPN11 | 0.01 | 0.49 | 20.00 | 0.79 | All matched targets |
| NR1H4 | 0.02 | 0.52 | 19.00 | 0.81 | All matched targets |
| CYP2C9 | 0.01 | 0.48 | 19.00 | 0.79 | All matched targets |
| ACHE | 0.01 | 0.50 | 19.00 | 0.80 | All matched targets |
| CYP2C19 | 0.01 | 0.46 | 19.00 | 0.77 | All matched targets |
| DRD2 | 0.01 | 0.49 | 19.00 | 0.79 | All matched targets |
| GSK3B | 0.01 | 0.51 | 19.00 | 0.81 | All matched targets |
| PRKCZ | 0.00 | 0.49 | 19.00 | 0.79 | All matched targets |
| HTR2A | 0.01 | 0.49 | 18.00 | 0.79 | All matched targets |
| CYP2D6 | 0.01 | 0.45 | 18.00 | 0.76 | All matched targets |
| FABP4 | 0.01 | 0.49 | 18.00 | 0.79 | All matched targets |
| CXCR2 | 0.00 | 0.50 | 18.00 | 0.80 | All matched targets |
| NOS2 | 0.00 | 0.51 | 18.00 | 0.81 | All matched targets |
| MMP1 | 0.00 | 0.51 | 18.00 | 0.81 | All matched targets |
| DRD4 | 0.00 | 0.48 | 17.00 | 0.78 | All matched targets |
| PRKDC | 0.00 | 0.50 | 17.00 | 0.80 | All matched targets |
| CTSB | 0.02 | 0.49 | 16.00 | 0.79 | All matched targets |
| SREBF2 | 0.01 | 0.47 | 16.00 | 0.77 | All matched targets |
| SLC6A4 | 0.00 | 0.47 | 16.00 | 0.77 | All matched targets |
| PARP1 | 0.00 | 0.49 | 16.00 | 0.79 | All matched targets |
| CHUK | 0.00 | 0.48 | 16.00 | 0.79 | All matched targets |
| MAPK9 | 0.00 | 0.50 | 16.00 | 0.80 | All matched targets |
| CYP1A2 | 0.01 | 0.44 | 15.00 | 0.75 | All matched targets |
| OPRD1 | 0.00 | 0.46 | 15.00 | 0.77 | All matched targets |
| ELANE | 0.00 | 0.49 | 15.00 | 0.79 | All matched targets |
| IKBKB | 0.00 | 0.47 | 15.00 | 0.77 | All matched targets |
| PTPN1 | 0.00 | 0.50 | 15.00 | 0.80 | All matched targets |
| FLT1 | 0.00 | 0.47 | 15.00 | 0.77 | All matched targets |
| SLC6A3 | 0.01 | 0.46 | 14.00 | 0.77 | All matched targets |
| NR1I2 | 0.01 | 0.48 | 14.00 | 0.78 | All matched targets |
| MAOA | 0.01 | 0.41 | 14.00 | 0.71 | All matched targets |
| NR1H3 | 0.00 | 0.45 | 14.00 | 0.76 | All matched targets |
| SHH | 0.00 | 0.47 | 14.00 | 0.78 | All matched targets |
| TGFBR1 | 0.00 | 0.45 | 14.00 | 0.75 | All matched targets |
| PDPK1 | 0.00 | 0.46 | 14.00 | 0.77 | All matched targets |
| ALK | 0.00 | 0.45 | 14.00 | 0.76 | All matched targets |
| MAOB | 0.01 | 0.42 | 13.00 | 0.73 | All matched targets |
| FAAH | 0.01 | 0.46 | 13.00 | 0.77 | All matched targets |
| FABP5 | 0.00 | 0.45 | 13.00 | 0.75 | All matched targets |
| INSR | 0.00 | 0.46 | 13.00 | 0.77 | All matched targets |
| MCHR1 | 0.00 | 0.44 | 13.00 | 0.75 | All matched targets |
| IDH1 | 0.01 | 0.45 | 12.00 | 0.76 | All matched targets |
| TTR | 0.01 | 0.48 | 12.00 | 0.78 | All matched targets |
| CES1 | 0.00 | 0.43 | 12.00 | 0.73 | All matched targets |
| RBP4 | 0.01 | 0.44 | 11.00 | 0.74 | All matched targets |
| VDR | 0.00 | 0.48 | 11.00 | 0.79 | All matched targets |
| TERT | 0.00 | 0.47 | 11.00 | 0.77 | All matched targets |
| GCK | 0.01 | 0.45 | 10.00 | 0.76 | All matched targets |
| ROCK1 | 0.00 | 0.43 | 10.00 | 0.74 | All matched targets |
| NR1I3 | 0.00 | 0.43 | 10.00 | 0.73 | All matched targets |
| CYP2A6 | 0.00 | 0.41 | 10.00 | 0.71 | All matched targets |
| PRKCE | 0.00 | 0.44 | 10.00 | 0.74 | All matched targets |
| FADS1 | 0.00 | 0.41 | 10.00 | 0.72 | All matched targets |
| CNR2 | 0.00 | 0.43 | 10.00 | 0.73 | All matched targets |
| PPARD | 0.00 | 0.45 | 9.00 | 0.75 | All matched targets |
| PLA2G2A | 0.00 | 0.43 | 9.00 | 0.74 | All matched targets |
| CETP | 0.00 | 0.43 | 9.00 | 0.74 | All matched targets |
| NAMPT | 0.00 | 0.47 | 9.00 | 0.77 | All matched targets |
| SLC27A4 | 0.00 | 0.41 | 9.00 | 0.71 | All matched targets |
| CYP17A1 | 0.01 | 0.43 | 8.00 | 0.73 | All matched targets |
| NPC1L1 | 0.00 | 0.43 | 8.00 | 0.73 | All matched targets |
| FDFT1 | 0.00 | 0.40 | 8.00 | 0.70 | All matched targets |
| CES2 | 0.00 | 0.39 | 8.00 | 0.69 | All matched targets |
| FABP3 | 0.00 | 0.41 | 8.00 | 0.72 | All matched targets |
| RIPK1 | 0.00 | 0.42 | 8.00 | 0.73 | All matched targets |
| GRIN2A | 0.00 | 0.44 | 8.00 | 0.75 | All matched targets |
| ACACB | 0.01 | 0.41 | 7.00 | 0.71 | All matched targets |
| HSD11B1 | 0.00 | 0.45 | 7.00 | 0.76 | All matched targets |
| FFAR1 | 0.00 | 0.43 | 7.00 | 0.74 | All matched targets |
| GC | 0.00 | 0.42 | 7.00 | 0.72 | All matched targets |
| NR1H2 | 0.00 | 0.43 | 7.00 | 0.73 | All matched targets |
| PSEN2 | 0.00 | 0.42 | 5.00 | 0.72 | All matched targets |
| PREP | 0.00 | 0.43 | 5.00 | 0.73 | All matched targets |
| PDE3B | 0.00 | 0.43 | 5.00 | 0.74 | All matched targets |
| PFKFB3 | 0.00 | 0.43 | 5.00 | 0.73 | All matched targets |
| ALOX12 | 0.00 | 0.41 | 5.00 | 0.71 | All matched targets |
| ENPP2 | 0.00 | 0.44 | 4.00 | 0.74 | All matched targets |
| ALPL | 0.00 | 0.42 | 4.00 | 0.72 | All matched targets |
| IL6ST | 0.00 | 0.40 | 4.00 | 0.70 | All matched targets |
| SHBG | 0.02 | 0.37 | 3.00 | 0.65 | All matched targets |
| CEL | 0.00 | 0.36 | 2.00 | 0.65 | All matched targets |
| CTSS | 0.00 | 0.34 | 2.00 | 0.62 | All matched targets |
| PYGL | 0.00 | 0.32 | 2.00 | 0.57 | All matched targets |
| HDAC8 | 0.00 | 0.39 | 2.00 | 0.69 | All matched targets |
| P2RX7 | 0.00 | 0.38 | 2.00 | 0.67 | All matched targets |
| ADH1C | 0.00 | 0.30 | 2.00 | 0.54 | All matched targets |
| ATP1A1 | 0.00 | 0.27 | 1.00 | 0.46 | All matched targets |
| AKT1 | 0.15 | 0.73 | 13.00 | 0.91 | Insulin-related targets |
| STAT3 | 0.04 | 0.63 | 12.00 | 0.86 | Insulin-related targets |
| PRKCZ | 0.03 | 0.63 | 12.00 | 0.86 | Insulin-related targets |
| PRKCD | 0.12 | 0.68 | 11.00 | 0.88 | Insulin-related targets |
| MAPK8 | 0.11 | 0.63 | 11.00 | 0.86 | Insulin-related targets |
| NOS3 | 0.07 | 0.66 | 10.00 | 0.87 | Insulin-related targets |
| PDPK1 | 0.03 | 0.59 | 10.00 | 0.83 | Insulin-related targets |
| PTPN11 | 0.01 | 0.58 | 9.00 | 0.82 | Insulin-related targets |
| PIK3CA | 0.01 | 0.56 | 9.00 | 0.80 | Insulin-related targets |
| PTPN1 | 0.01 | 0.56 | 8.00 | 0.80 | Insulin-related targets |
| INSR | 0.01 | 0.53 | 7.00 | 0.78 | Insulin-related targets |
| MAPK9 | 0.00 | 0.54 | 7.00 | 0.79 | Insulin-related targets |
| GSK3B | 0.00 | 0.53 | 7.00 | 0.78 | Insulin-related targets |
| PRKCE | 0.00 | 0.53 | 6.00 | 0.78 | Insulin-related targets |
| IKBKB | 0.00 | 0.51 | 6.00 | 0.76 | Insulin-related targets |
| PPARA | 0.27 | 0.58 | 5.00 | 0.82 | Insulin-related targets |
| ACACB | 0.11 | 0.39 | 2.00 | 0.61 | Insulin-related targets |
| NR1H2 | 0.02 | 0.44 | 2.00 | 0.68 | Insulin-related targets |
| NR1H3 | 0.02 | 0.40 | 2.00 | 0.62 | Insulin-related targets |
| PYGL | 0.00 | 0.28 | 1.00 | 0.37 | Insulin-related targets |

The values were rounded up to 2 decimal places
